# Electrified thermochemical reaction engineering with metamaterial reactors

**DOI:** 10.1126/sciadv.aeg7217

**Published:** 2026-09-02

**Authors:** Ariana B. Höfelmann, Chenghao Wan, Calvin H. Lin, Dolly L. Mantle, Zhennan Ru, Connor Cremers, Kesha Tamakuwala, Matthew W. Kanan, Juan Rivas-Davila, Jonathan A. Fan

**Affiliations:** ^1^ Department of Electrical Engineering, Stanford University, Stanford, CA, USA.; ^2^ Department of Mechanical Engineering, Stanford University, Stanford, CA, USA.; ^3^ Department of Material Science Engineering, Stanford University, Stanford, CA, USA.; ^4^ Department of Chemistry, Stanford University, Stanford, CA, USA.

## Abstract

The electrification of thermochemical reactors presents an opportunity to not only decarbonize high-grade heat generation but also to customize heating and heat transfer processes in a manner that facilitates process intensification and enhanced conversion. We present metamaterial reactors that use the high-frequency magnetic induction of axially tailored metamaterial baffles to produce customized axial heating profiles. The baffle consists of a combinatorial sequence of two types of lattices each with distinctive heating properties, and the sequence can be inverse designed around the reaction engineering properties of the reactor to produce temperature profiles tailored for a given reaction, flow condition, and maximum temperature. We use these metamaterial reactors to enhance the temperature uniformity in a set of packed bed flow reactors, each driving the reverse water gas shift reaction under different maximum temperature and flow conditions, demonstrating systematic increases in conversion compared to uniformly heated systems. These concepts encapsulate new opportunities in electrified thermochemical reaction engineering in which electrified powering is codesigned with reaction and heat transfer processes to enhance chemical conversion.

## INTRODUCTION

Endothermic gas phase thermochemistry is foundational to many core processes in the chemicals industry (*1*). For these processes, control over the volumetric temperature profile and heat transfer rates within a chemical reactor determines critical performance parameters including thermodynamic conversion, reaction kinetics, and energy flux to reactant gases and catalyst materials (*2*). The ideal temperature profile for a given system depends on the reaction and ranges from isothermal, which maximizes conversion for elementary endothermic reactions (*3*), to highly specific temperature profiles that enhance selectivity in processes featuring parasitic side reaction pathways (*4*, *5*). Conventional methods for reactor heating include the use of combustion furnaces and heat exchange networks (*6*), which are mature technologies but ultimately suboptimal in the context of spatial temperature control.

The development and implementation of new heating and heat transfer concepts that can improve volumetric temperature profile management have played a featured role in advancing reactor performance, and various concepts in this vein have been proposed. Multistage reactors use serial combinations of heating and reaction sections to improve axial temperature control (*7*–*9*). Microreactors use reductions in reactor size to enable enhanced heat transfer with an external reservoir (*10*–*12*). Structured reactors use baffles with tailored thermal properties, in particular, those with enhanced thermal conductivity, which mitigate parasitic temperature gradients within the reactor (*13*–*15*). While these concepts have pushed the capabilities of reactors toward more ideal operating conditions, there remains substantial distance between physical implementation and theoretical optimal performance due to capability, complexity, cost, and scaling.

The electrification of heat generation presents a qualitatively new route to clean energy delivery within thermochemical reactors that has the potential to overcome many of these limitations. With electrification, structures serving as resistors or susceptors are directly and internally heated using electric currents or electromagnetic waves, presenting simplifications to heating infrastructure and new avenues for tailored heating based on resistor and susceptor design (*16*, *17*). Concepts based on electromagnetic induction, which will be the focus here, present a particularly attractive route to electrified heating due to the volumetric nature of generated heating profiles, galvanic isolation between the reactor and power source, transduction of high voltages from a drive coil to high currents in a susceptor, and the ability to be highly efficient when properly designed (*18*–*21*).

Induction heating has been previously implemented as a means for heating catalyst-coated nanoparticles (*22*) with customized heating responses based on composition (*23*). Other studies have considered the induction heating of metal tubes (*24*, *25*), which enables retrofitting of tube-based reactor schemes but which have limited heating efficiencies and heat transfer properties. Recent work has also explored the heating of uniform, volumetric baffles packed with catalysts, indicating their potential as modular and customizable heating susceptors supporting volumetric heating profiles (*26*, *27*). The bulk of these studies focus on reactor configurations supporting axially uniform heating, and a major and underexplored opportunity is the tailoring of these axial heating profiles to improve reactor performance.

**Fig. 1.**
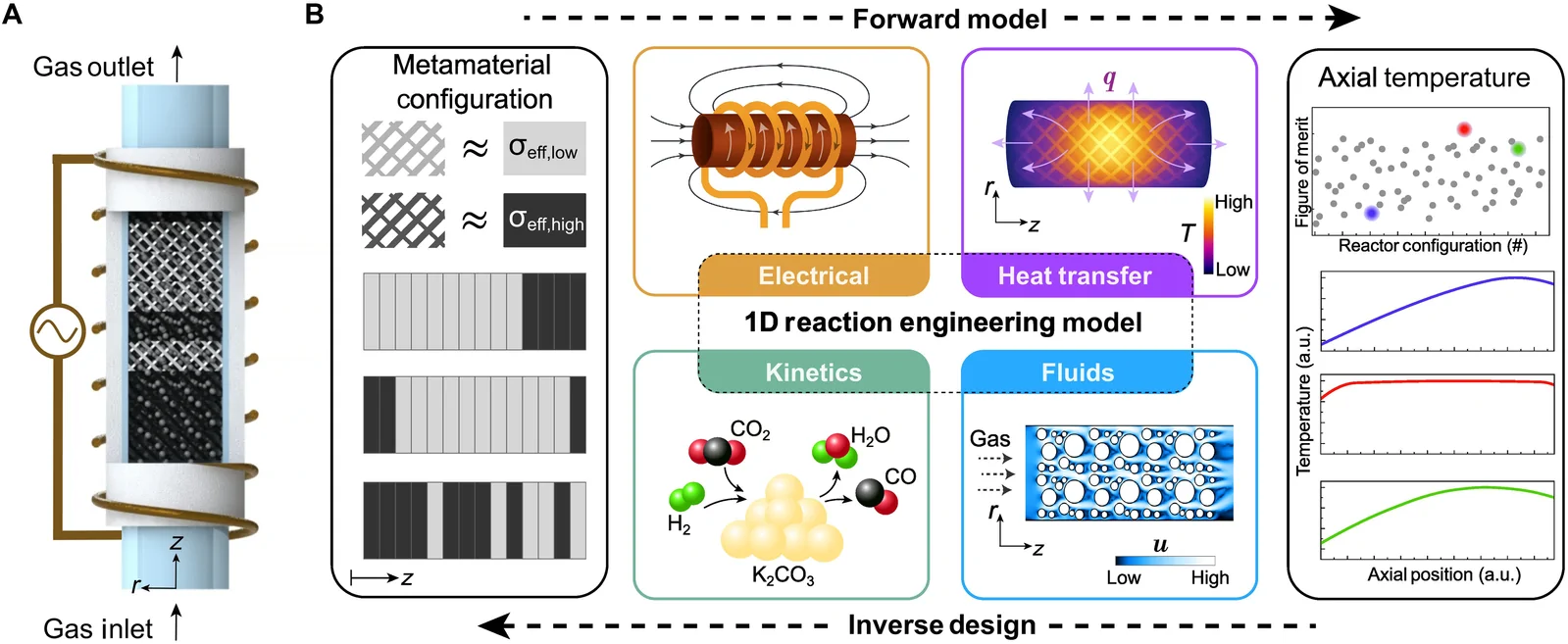
Overview of metamaterial reactors with axial tailoring. (**A**) Schematic of an inductively heated metamaterial reactor with axial tailoring. A sequence of two types of open cell lattice baffle sections are heated with a helical magnetic coil. (**B**) The axial heating profile is specified by the unique sequence of metamaterial sections in the reactor, which are characterized by one of two effective electrical conductivity values. A 1D reaction engineering model that accounts for heating, heat transfer, mass transfer, and reaction kinetics serves as a forward model that maps the axial heating profile to the axial temperature profile. This model can be used for inverse design in which a susceptor sequence is identified that yields a temperature profile that maximizes a desired figure of merit (FOM).

We present inductively heated metamaterial reactors that implement customized axial heating profiles codesigned with reaction conditions to enhance reactor performance. The reactor, depicted in Fig. 1A comprises a defined sequence of two types of metamaterial baffle sections that dissipate heat via eddy current generation when inductively heated with a helical magnetic coil. Each baffle section consists of an open cell lattice packed with fixed bed catalyst particles wherein the high thermal conductivity and large surface area of the lattice facilitates conductive heat transfer to the catalyst. We refer to our reactor baffle sections as metamaterials because the open cell lattices can be macroscopically described in the effective medium limit to have homogenized electrical conductivities (*28*), and we consider baffle sections to have one of two effective electrical conductivity values in our metamaterial system. The axial heating profile is tailored by specifying a unique sequence of baffle sections along the reactor, and discretization ensures that only two types of baffle sections need to be manufactured, independent of the desired heating profile. The spatial resolution of the heating profile is set by the width of a single baffle section and far exceeds the spatial resolution specified by multistage reactor architectures.

In this implementation of metamaterial reactors, the axial heating profile is explicitly tailored to produce an optimal temperature profile given a particular reaction, catalyst, maximum operating temperature, and gas flow rate. Such hardware specificity is typical in the chemicals industry, where reactors are often designed to operate under particular conditions in conjunction with upstream and downstream processes. Our approach to designing around the nontrivial relationship between the heating and temperature profile within a flow reactor is outlined in Fig. 1B. The mapping of heating profile to temperature profile, given a specific reaction condition and susceptor configuration, is specified with a multiphysics forward model, which combines energy balance, mass balance, and reaction kinetics relationships that collectively account for conductive and convective heat transfer, reaction enthalpy, and induction heating in the reactor. With an established forward model, inverse design can be performed in which a specific metamaterial susceptor configuration is identified based on a desired temperature profile. Inverse design can be performed using numerical optimization of the discrete susceptor system (*29*, *30*), and it can also be achieved by evaluation of all susceptor section combinations pending the availability of a fast forward model solver. These concepts present new opportunities in electrified thermochemical reaction engineering, in which the high degree of spatial heating control enabled by electrification is combined with reaction engineering-centric system design principles to enhance reactor performance.

Metamaterial reactors can be adapted to a wide range of reaction processes, reactor types, and desired temperature profiles. In this study, we investigate endothermic gas phase reactions in a fixed bed flow reactor as a model system and specifically focus on the reverse water gas shift (RWGS) reaction (CO_2_ + H_2_ ↔ CO + H_2_O) using a supported potassium carbonate catalyst that suppresses the methanation side reaction (*31*, *32*). RWGS has been an emergent reaction of interest for the circular carbon economy because it uses carbon dioxide as a feedstock (*33*, *34*). Our objective centers around the design of a set of reactors, each operating at a specific maximum temperature and gas flow rate and each requiring a distinct combination of metamaterial baffles, to be as close to isothermal as possible to maximize conversion. Our forward model consists of one-dimensional (1D) steady state governing equations relating reactor temperature and conversion to induction heating response and is fit with experimental reaction data to ensure generalization and accuracy. For our lab-scale demonstration, each metamaterial baffle section has a width of 12.5 mm and a radius of 19 mm, and the total reaction zone comprises 14 sections and is approximately 175 mm long. Our specification of a small section width relative to its diameter ensures high spatial resolution heating control.

## RESULTS AND DISCUSSION

### Metamaterial susceptor design and implementation

**Fig. 2.**
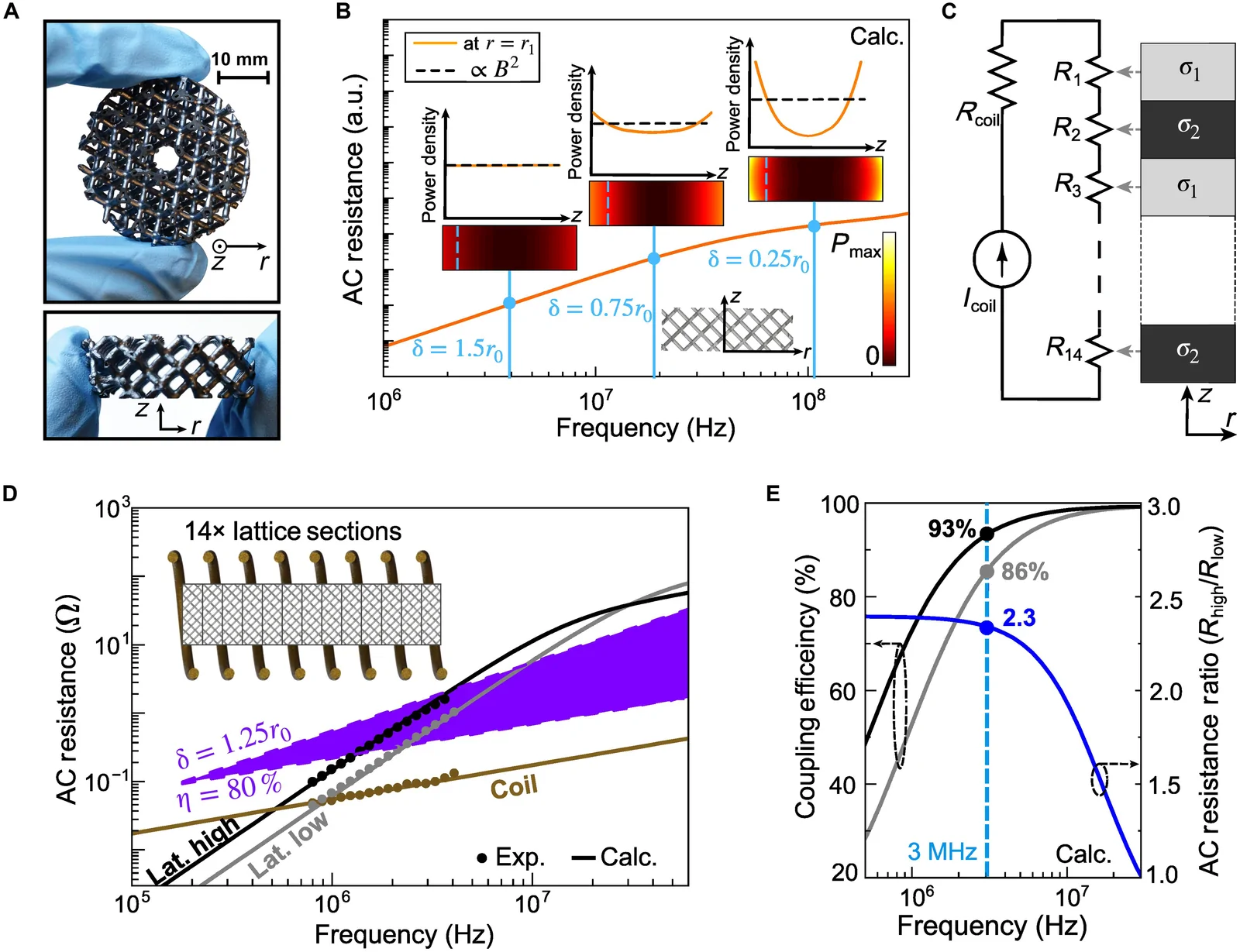
Inductive heating properties of metamaterial lattice sections. (**A**) Images of the front (top) and side (bottom) view of a SiSiC lattice section. (**B**) AC resistance of a single metamaterial section in a uniform magnetic field as a function of frequency. The insets show images and axial linescans of the cross sectional power dissipation profiles at different $\delta /r_{0}$ ratios. For $\delta /r_{0}<1.25$, the power profile experiences finite-sized effects and is axially nonuniform. For $\delta /r_{0}>1.25$ (i.e., the large skin depth limit), the axial power profile is axially uniform and proportional to $B^{2}$. (**C**) Equivalent circuit model for our metamaterial reactor in a uniform magnetic field in the large skin depth limit. $I_{\text{coil}}$ and $R_{\text{coil}}$ represent the current driving through the magnetic coil and dissipation in the coil, respectively. Each of the 14 sections is represented by a resistor $R_{i}$ that has one of two possible values depending on the susceptor type. (**D**) Plot of AC resistance as a function of frequency, delineating the susceptor design regime (purple) that is bounded by the large skin depth limit (top line) and the minimum coupling efficiency metric (bottom line). Also plotted are the AC resistances of the helical magnetic coil and two candidate SiSiC susceptor types (14 total sections for each curve). The dots are experimental data and the solid lines are fitted curves. (**E**) Plot of coupling efficiency and AC resistance ratio as a function of frequency for the two SiSiC susceptor types shown in (D). The dashed line at 3 MHz delineates our chosen operating frequency.

In this section, we focus on developing a design framework for the metamaterial susceptor sections. Multiple criteria must be met: the efficient and volumetric heating of the complete metamaterial section sequence, large differential heating between the two metamaterial section types, and decoupled electromagnetic interactions between neighboring sections, which simplifies thermal engineering of the full metamaterial sequence. We will focus on the utilization of additively manufactured reaction bonded silicon carbide (SiSiC) lattice sections, which are ideal for our application because SiSiC is chemically inert, refractory, and has a low thermal expansion coefficient that is matched with the catalyst support. SiSiC additionally has an electrical conductivity that can be tuned by varying the amount of excess silicon loading (*35*–*37*). As such, the electromagnetic properties of the metamaterial sections can be tuned without modifying the lattice geometry, ensuring that fluid dynamics and catalyst loading properties are the same for the two section types. Images of an additively manufactured metamaterial section are shown in Fig. 2A. A cubic cell lattice with a 5-mm pitch and 1-mm-diameter struts is specified to support uniform effective electrical conductivity properties and ease of catalyst particle loading into the baffle.

We first identify induction heating regimes for susceptor sections that support volumetric heating and decoupled electromagnetic interactions with neighboring sections. Our approach will build on prior work on the induction heating of homogeneous open cell lattice susceptors in the infinite cylinder limit, where it was found that open cell lattices have effective electrical conductivities ($\sigma_{\text{eff}}$) that could be described by the Lemlich limit (*38*). It was further found that volumetric heating with high heating efficiencies occurs when the susceptor skin depth $\delta =1/\sqrt{\mu_{0}\pi f\sigma_{\text{eff}}}\approx r_{0}/2$, where f is the induction frequency, $r_{0}$ is the susceptor radius, and $\mu_{0}$ is the magnetic permeability of free space. Our susceptor sections are distinct from infinite cylinder systems as they can feature strong finite-size electromagnetic effects, leading to modified heating regimes. Calculated AC resistance from an individual susceptor section ($\sigma_{\text{eff}}=100\;S/m$) with experimental dimensions as a function of frequency, together with power dissipation profiles within the section at three frequencies, are presented in Fig. 2B, assuming a uniform external magnetic field. We find that when $\delta /r_{0}<1.25$, the metamaterial section heating profile is axially nonuniform and has heating properties that are strongly influenced by the presence of neighboring sections (see section S1.1). However, when $\delta /r_{0}>1.25$, individual metamaterial sections feature heating profiles with minimal finite-size effects, high axial uniformity, and insensitivity to the presence of neighboring sections. Furthermore, the total power dissipation in an individual susceptor section can be modeled analytically and its axial power dissipation profile is proportional to the square of the external magnetic field magnitude. We therefore choose to specify both susceptor types to operate within this large skin depth regime.

The metamaterial reactor can be subsequently modeled by an equivalent circuit comprising a series of decoupled resistors (Fig. 2C). The model includes a current source ($I_{\text{coil}}$), a coil resistor ($R_{\text{coil}}$), which represents ohmic dissipation in the magnetic coil itself, and 14 resistors representing each of the metamaterial sections ($R_{1,\ldots ,14}$). The resistors each represent one of the two metamaterial section types, have magnitudes of $R_{\text{high}}$ and $R_{\text{low}}$ depending on whether they support relatively high or low power dissipation, respectively, and have no interdependence due to the decoupling of electromagnetic interactions between sections and their neighbors. The total amount of power dissipated in the susceptor sections is $I_{\text{coil}}^{2}R_{\text{sus}}$, where $R_{\text{sus}}=\sum_{1}^{14}R_{i}$ includes all susceptor sections, and the coupling efficiency (η) represents the fraction of power dissipated in the susceptor sections relative to the power inputted into the magnetic coil and is $R_{\text{sus}}/\left(R_{\text{sus}}+R_{\text{coil}}\right)$. We additionally specify the AC resistance ratio, $R_{\text{high}}/R_{\text{low}}$, which represents the relative power dissipation between the two section types. See section S1.2 for details.

Our skin depth criteria and circuit picture can be used to specify operating frequencies and susceptor effective conductivity values that balance coupling efficiency with AC resistance ratio. As a design choice, we set the minimum η of our system to be 80%, which is enforced when a reactor configuration comprising all low power dissipating section types yields η=80%. Lowering the minimum coupling efficiency threshold can yield larger AC resistance ratios and higher levels of thermal engineering in the reactor, which will be explored later in this study. To visualize design regimes that satisfy our posed constraints, we plot in Fig. 2D design bounds shaded in purple, the coil AC resistance, and the AC resistances of two candidate susceptor types, discussed below. The coil AC resistance is based on experimental characterization of our nine turn copper helical coil. The upper design boundary specifies the condition $\delta /r_{0}=1.25$ and represents the ideal AC resistance of the more conductive susceptor type, while the lower design boundary specifies the minimum coupling efficiency criteria and represents the ideal AC resistance of the lower conductivity susceptor type. These bounds indicate that there is a minimum operating frequency for metamaterial reactors, in our case in the hundreds of kilohertz range for a lab-scale system. Furthermore, increasing the operating frequency leads to a larger gap in AC resistance between the upper and lower bound curves, providing the potential for enhanced AC resistance ratios.

The susceptor AC resistance curves are each based on the experimental characterization of 14 sections of a given lattice type, with each type representing a distinctive level of silicon loading specified by our manufacturing process. Curve fitting from experimentally measured AC resistance values indicate that the $\sigma_{\text{eff}}$ of the high- and low-conductivity lattices are 115 and 48 S/m, respectively (see section S1.3). Together with experimentally measured AC resistance values of the helical magnetic coil, the coupling efficiencies and AC resistance ratios can be calculated as a function of frequency (Fig. 2D). These plots visualize the fundamental trade-off between coupling efficiency and AC resistance ratio. We select an operating frequency of 3 MHz, which sets the AC resistance of high power dissipating lattice type to the condition $\delta /r_{0}=1.25$, coupling efficiencies to 14 sections of the high and low conductivity susceptor types to be 93 and 86%, respectively, and the AC resistance ratio to be 2.3.

Up to this point, we have assumed that the magnetic field is axially uniform, which is a typical approximation used to describe high-aspect-ratio helical coils and which presents a simplifying limit for analyzing our metamaterial concept. In practice, the magnetic field is often not uniform, and in our case, axial field inhomogneities are introduced with our utilization of a finite-length helical magnetic coil with uniform turn spacing. An examination of the analytic magnetic field profile for our coil shows that the field is approximately radially uniform within the coil core but axially varies, yielding a general form of $B\left(z\right)=I_{\text{coil}}\;f\left(z\right)$. For such magnetic fields and susceptors operating in the large skin depth regime, we can readily modify our circuit description of susceptor heating by multiplying the susceptor resistance values by ${|f\left(z\right)|}^{2}$ and a normalization constant. We use this modified susceptor resistance picture moving forward in the design of our experimental metamaterial reactors. More details are provided in sections S1.4 and S1.5.

We note that while our metamaterial platform is constrained to only two types of susceptor sections, it can exhibit a level of temperature control that is commensurate with a more expressive platform supporting continuously varying effective conductivities. As a demonstration, we numerically simulate the temperature profile of baffles with continuously varying conductivity profiles together with metamaterial sequences designed to closely match these temperature profiles. In the limit where the section width is significantly smaller than its radius, an effective medium description can be formulated in which small sets of susceptor sections can be grouped together and collectively described with continuous effective conductivity values that are a function of susceptor grouping sequence. A more detailed analysis of this this hierarchical metamaterial description is presented in the section S1.6.

### Metamaterial reactor implementation

**Fig. 3.**
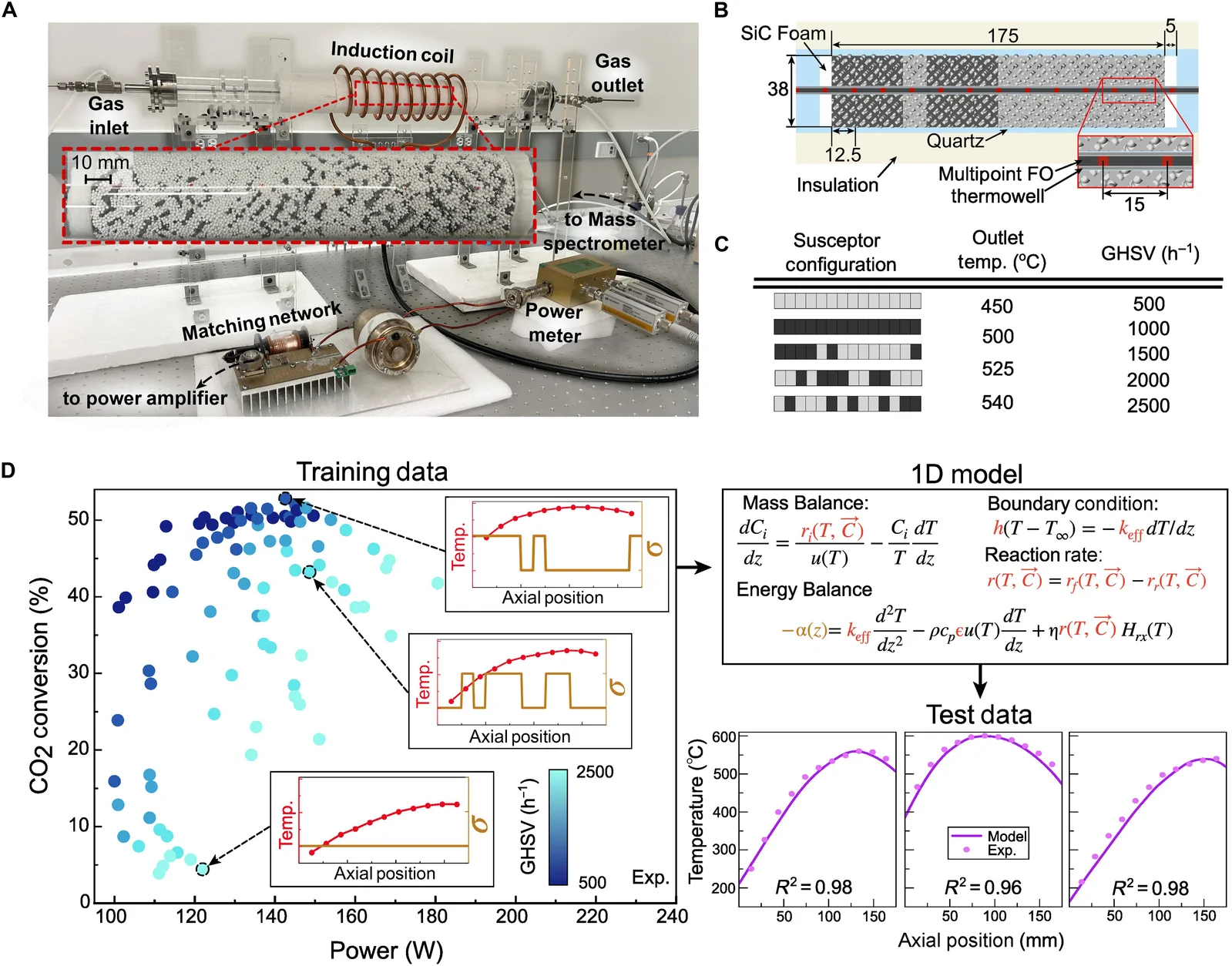
Experimental metamaterial reactor implementation and calibration. (**A**) Image of the reactor setup, including the inductively heated reactor and associated power electronics and gas lines. The inset shows the metamaterial baffle packed with catalyst particles. (**B**) Schematic of the reaction zone, together with the configuration and placement of the fiber optic temperature probe. All dimensions are in mm. (**C**) Table summarizing combinations of susceptor configurations, outlet temperatures, and gas flow rates used for 1D model calibration. (**D**) Experimental visualization of the calibration data and results. The training data (left) is collected from 90 unique reactor configurations as specified in (C) and is used to fit parameters in our 1D model (red terms, top right). Our calibrated model produces axial temperature profiles that fit our test datasets with high accuracy, as shown in three representative examples (bottom right). The fitted and experimental maximum temperatures are set to be equal as a normalization condition to the fitting process.

With our susceptor design framework, we experimentally calibrate and implement our metamaterial reactor for the RWGS reaction. Our experimental setup is shown in Fig. 3A. The reactor body comprises a 39-mm-diameter quartz tube surrounded by 12.5-mm-thick zirconia insulation and our helical copper coil, loaded with our lattice sections and 1-mm-diameter catalyst pellets comprising potassium carbonate supported on mesoporous alumina. Our baseline configuration consists of 14 sections of the low electrical conductivity SiSiC lattice. Inverse designed configurations are implemented using sections of both high and low electrical conductivity SiSiC. Mass flow controllers at the reactor inlet specify the composition and flow rate of the reactant gases, and products are characterized using a mass spectrometer at the outlet. The reactor is powered using a signal generator coupled to a broadband power amplifier and matching network, and a power meter is used to quantify the amount of power fed into the helical coil. More details pertaining to the setup are in the Materials and Methods section below, and in section S2.1. Axial temperature measurements within the reactor are taken using a 15 channel fiber optic sensor (15-mm channel spacing) placed through the radial center of the reaction zone (Fig. 3B). The 15 mm resolution was experimentally verified to accurately capture the axial temperature profile (details are in section S2.2).

To calibrate the 1D reaction engineering model describing our reactor, we experimentally collect axial temperature and conversion data for a range of susceptor section sequences, outlet temperatures, and inlet gas flow conditions (Fig. 3C). The outlet temperature range is 450° to 540°C, which features a wide range of reaction kinetics and chemical conversion behavior as determined from prior study (*18*, *31*), and the gas hourly space velocity (GHSV) range is 500 to 2500 hour^−1^, which ensures laminar flow as well as negligible pressure drop and shear effects (see section S2.3). Our 1D reaction engineering model assumes a pseudo-homogeneous reactor in which the catalyst-loaded susceptors are described with effective thermal and electrical properties, and we neglect radial temperature gradients, which is a reasonable approximation for lab scale reactors (*39*–*41*). This approximation is further justified with multiphysics simulations performed in COMSOL, which show negligible radial temperature gradients for the system described here. In addition, the radial Biot number is calculated to be much less than 1, indicating a high degree of radial temperature uniformity. Details can be found in sections S3.1 and S3.2. Our 1D model accounts for heat transfer via conduction and convection, and at the temperatures considered here, radiative effects are negligible. The coupled energy balance-mass balance expressions are

$$k_{\text{eff}}\frac{d^{2}T}{{\mathrm{dz}}^{2}}-\rho c_{p}\epsilon u\left(T\right)\frac{\mathrm{dT}}{\mathrm{dz}}-\eta r\text{(}T,C\text{)}H_{\mathrm{rx}}\left(T\right)+\alpha \left(z\right)=0$$ (1)

$$\frac{{\mathrm{dC}}_{i}}{\mathrm{dz}}=\frac{\nu_{i}r_{i}\text{(}T,C\text{)}}{u\left(T\right)}-\frac{C_{i}}{T}\frac{\mathrm{dT}}{\mathrm{dz}}$$ (2)

$k_{\text{eff}}$ is the thermal conductivity, ρ is the density of the gas moving through the reactor, $c_{p}$ is the heat capacity of the gas moving through the reactor, ϵ is the effective porosity of the packed susceptor, *u* is the gas superficial velocity, η is the catalyst effectiveness factor, r(T,C) is the reaction rate, $H_{\mathrm{rx}}\left(T\right)$ is the heat of reaction, $C_{i}$ is the molar concentration of species *i*, C is the vector of reactant and product concentrations and $\nu_{i}$ is the stoichiometric coefficient of species *i*. α(z) is the axial power density from metamaterial susceptor heating, and it is based on the formalism developed in the previous section. The reaction rate is specified to be elementary and reversible with $r=r_{\text{forward}}\text{(}T,C,A_{f},E_{f}\text{)}+r_{\text{reverse}}\text{(}T,C,A_{r},E_{r}\text{)}$, where $A_{i}$ are the preexponential factors and $E_{i}$ are the activation energies. Convective boundary conditions are applied at the inlet and outlet, where the heat transfer coefficient *h* is defined with the expression $h\left(T-T_{\infty }\right)=-k_{\text{eff}}dT/dz$.

A summary of our data collection and fitting process is shown in Fig. 3D. A total of 100 data points are collected in steady-state reactor operation conditions, from which 90 of the points are used as fitting data and 10 of the points are used for model testing. The α(z) profile is determined from the analytic helical coil magnetic field profile, experimental AC resistance characterization of the susceptor sections, and measured input power into the coil. The colored variables in the 1D model expressions (r(T,C), ϵ, $k_{\text{eff}}$, and h) are systematically determined using least squares data fitting. The kinetics parameters are fit independently from the heat transfer parameters, using the collected experimental conversion and axial temperature profiles, using Eq. 2. Once the activation energy and prefactor were determined, they were used to train our 1D pseudo-homogeneous model described by the energy balance above. More details of the parameter extraction can be found in section S3.3.

In using the model, we explicitly set the fitted maximum temperature (i.e., $T_{\text{max}}$) value in a given profile to match with the experimental maximum temperature by making slight adjustments to the input power magnitude. We add this normalization condition to account for the fact that even with the known amount of power inputted into the coil, the absolute amount of power dissipated in the reactor is difficult to accurately quantify due to unaccounted parasitic field coupling to the environment and slight variations in susceptor conductivity within the experimental pool of susceptor sections. While this condition limits our ability to use the fitted model in an open loop manner, its impact on model-driven inverse design is minimal, as small variations in absolute inputted power lead to lateral shifts in the temperature profile without significant modification to its detailed shape as demonstrated in section S3.4. The fit model is capable of accurately generalizing given a set maximum temperature value, and with the testing data, our model predicts temperature curves within the reactor with an average *R*^2^ value of 0.97 and an average root mean square error of 14.6. We also perform cross-validation using different test and training sets, which indicate consistent model performance (see section S3.5). Three representative examples of predicted and actual temperature profiles from the testing data are shown in the lower right of Fig. 3D and show good agreement.

**Fig. 4.**
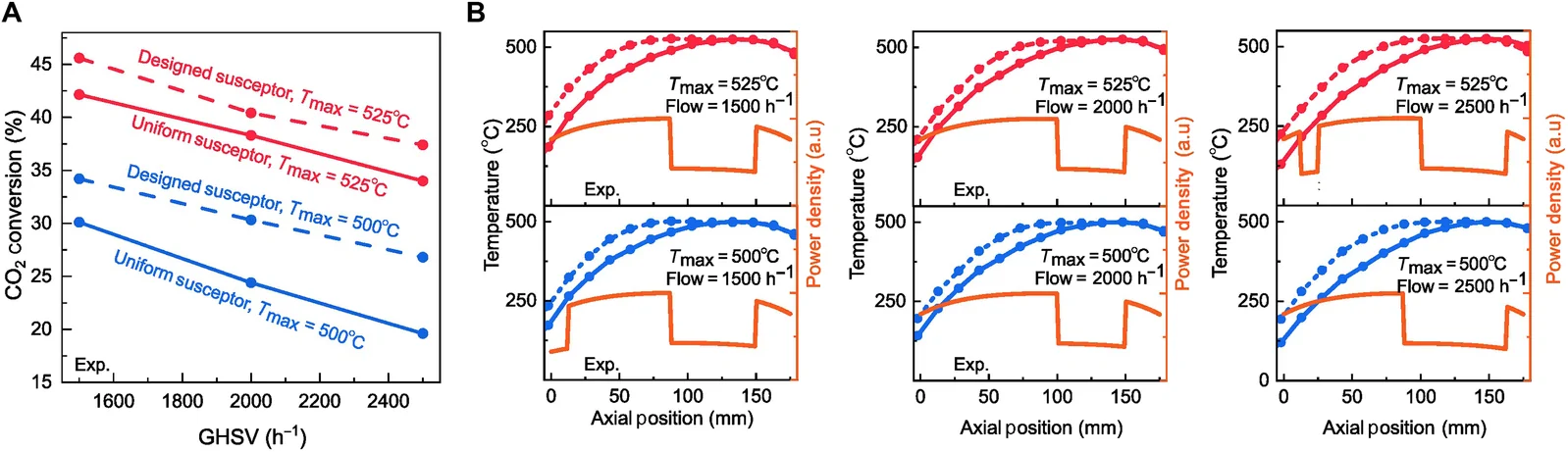
Experimental performance of metamaterial reactors with axial tailoring, configured for the RWGS reaction. (**A**) CO_2_ conversion as a function of GHSV for uniform susceptor metamaterial reactors (solid lines) and inverse designed metamaterial reactors (dashed lines) designed to operate at two different maximum temperatures, 500°C (blue) and 525°C (red). Conversion with the tailored metamaterial reactor increases by 2 to 6% relative to the uniform reactor for each case. (**B**) Temperature profiles of the uniform and inverse designed metamaterial reactors for the six conditions in (A), together with the power density profile of the optimized inverse designed susceptor sequence.

With a fully defined model, we perform inverse design of the metamaterial stack with the aim of maximizing CO_2_ conversion given a set flow condition and maximum temperature. With our ability to evaluate our 1D model at high speeds, we compute the temperature profile and conversion percentage for all 2^14^ possible metamaterial susceptor configurations and search for the reactor configuration that maximizes the figure of merit (FOM) defined as $\text{(}\%{\text{CO}}_{2,i}-\%{\text{CO}}_{2,u})/\text{(}T_{\text{max}}-T_{i,\text{outlet}}\text{)}$. $\%{\text{CO}}_{2,i}$ is the conversion for the $i^{th}$ susceptor configuration, $\%{\text{CO}}_{2,u}$ is the conversion of a uniform reactor at the same conditions, and $T_{i,\text{outlet}}$ is the outlet temperature of the $i^{th}$ susceptor configuration. The terms in the denominator explicitly minimize temperature gradients at the reactor outlet, and they are added to account for the sensitivity of conversion to outlet temperature gradients and improve the robustness of the susceptor design. A comparison of FOMs can be found in section S3.6. The experimental performance of metamaterial reactors designed for six different maximum temperature and gas flow rate combinations are presented in Fig. 4 together with uniform susceptor references. We observe an increase in experimental CO_2_ conversion by 2 to 6% for every tested condition, which is statistically significant as confirmed by a two-sample *t* test (see section S3.7). This increase in conversion follows an enhancement in temperature uniformity observed for all reactor configurations (Fig. 4B), where the difference between the minimum and maximum temperatures within the central region of the reaction zone (i.e., axial positions 25 to 150 mm) decreased by approximately 100°C for every reactor configuration relative to the uniform susceptor references.

We observe that the optimal metamaterial sequences, shown in Fig. 4B, all generally exhibit selective heating at the inlet and outlet with enhanced heating specifically at the reactor inlet. The need for selective heating at the inlet and outlet is attributed in part to thermal conduction losses, which are dominant at the ends of the reactor due to enhanced heat losses at the open air boundary of the susceptor. Additional heating at the inlet is necessary due to thermal convection losses, which are most significant at the inlet where the incoming gases are cold. It is also due to enhanced reaction consumption near the inlet, where reactant concentrations are highest. Each of these heat loss mechanisms are a function of both temperature and flow rate, which necessitates our current approach of tailoring the heating profile for a unique set of operating conditions through careful inverse design.

**Fig. 5.**
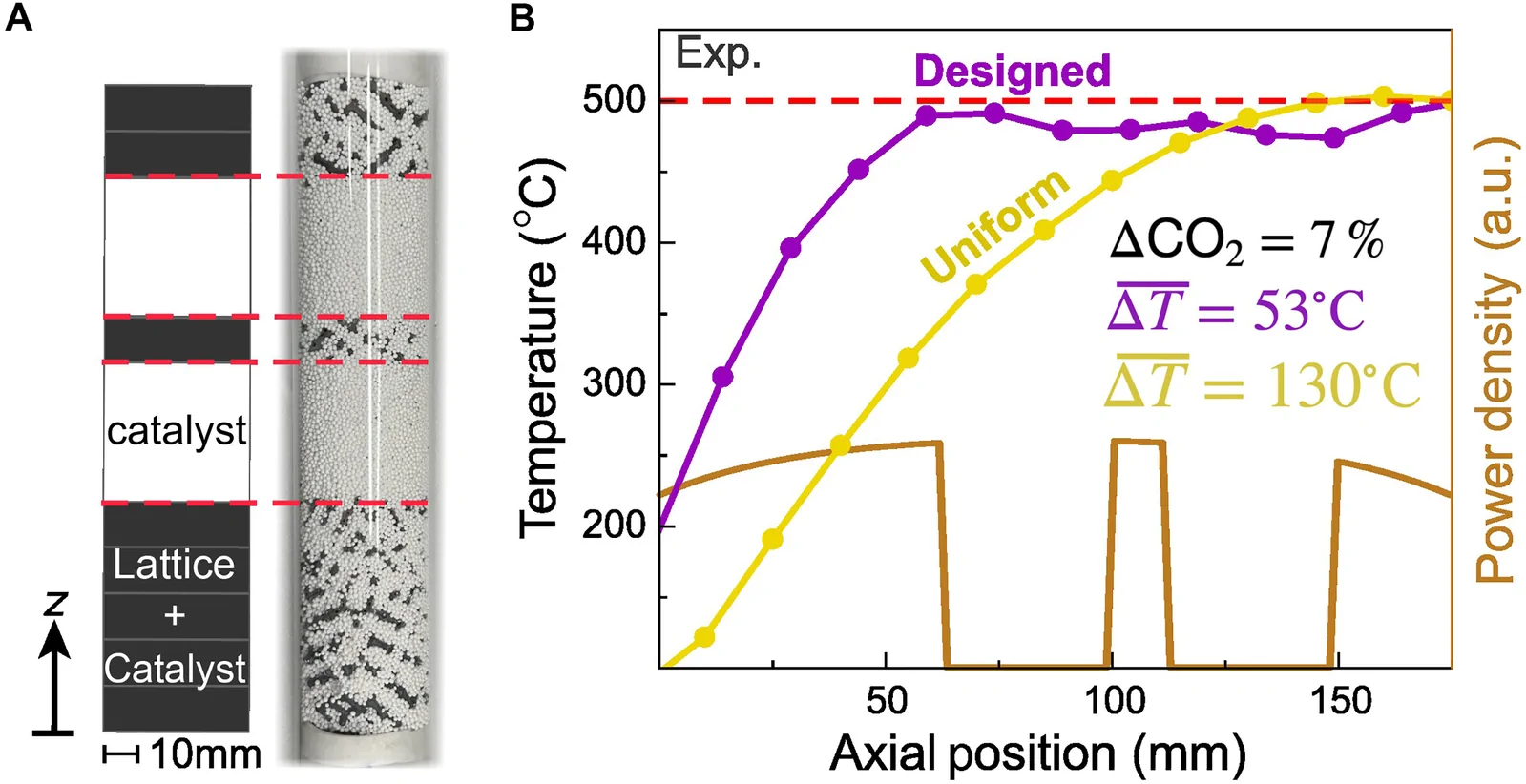
Metamaterial reactors with enhanced AC resistance ratio. (**A**) Schematic and picture of a metamaterial reactor system with an infinite AC resistance ratio. (**B**) Experimental axial temperature profiles for the inversely designed reactor configuration (purple) shown in (A) and a uniformly heated reactor reference (yellow), together with the power density profile of the inverse designed susceptor sequence.

In the final part of our analysis, we investigate how enhanced thermal profile engineering can be achieved with susceptors featuring larger AC resistance ratios. In particular, reactors with larger resistance ratios can unlock more extreme heating profiles, including those featuring higher relative power dissipation at the reactor inlet, thereby further improving axial temperature profile uniformity. We experimentally implement a proof-of-concept metamaterial reactor system with a resistance ratio of infinity, in which one of the reactor section types comprises the lattice susceptor packed with catalyst while the other section type comprises only catalyst without a susceptor (Fig. 5A). We perform susceptor inverse design with a modified 1D model using the same design procedure as for the low AC resistance ratio reactors (see section S4.1 for more details).

The resulting axial temperature profile and power dissipation profile of our reactor, designed for a GHSV of 2500 hour^−1^ and maximum temperature of 500°C, is shown in Fig. 5B together with a uniformly heated metamaterial reactor as a reference. See section S4.2 for details on the experimental set up. The optimized reactor shows that the temperature at the inlet reaches the target maximum temperature setpoint in much shorter axial length scales compared to the uniformly heated case, and that the temperature profile is generally more axially uniform, resulting in a relative CO_2_ conversion increase of 7%. The average axial temperature difference within the reactor, which is defined as the axially averaged difference between the setpoint temperature and measured temperature, is a factor of two smaller in the inversely designed reactor compared to the uniformly heated reactor. We note that the reactor as implemented displays more axial temperature fluctuations compared to low AC resistance ratio reactors, as the more extreme heating profile yields hot and cold spots in the reactor. We anticipate that further improvements in temperature uniformity in the large AC resistance ratio limit can be achieved with higher spatial resolution susceptor configurations.

### Outlook

In this study, we demonstrate and validate the concept of inductively heated metamaterial thermochemical reactors that feature axial temperature profile control based on inverse design of the metamaterial susceptor electrical conductivity profile. We identify design regimes for susceptor sections that enable volumetric heating, high coupling efficiencies, and electromagnetic decoupling between sections. We further combine this design methodology with a calibrated 1D reaction engineering model to identify susceptor section sequences that enhance temperature uniformity and conversion within flow reactors. This codesign of electrified heating with reaction engineering reflects the potential for electrified chemical conversion systems to push reactor capabilities beyond conventional limits, made possible by the unique ability for wave-based heating to deliver energy to precise locations within a volumetric body.

The design flexibility built in to our metamaterial susceptor framework can facilitate a direct pathway toward scaling. In particular, susceptor sections featuring radially varying effective electrical conductivity profiles can be implemented to support uniformly volumetric heating (*21*), eliminating parasitic radial gradients within sections, and enabling a 1D description of the axial heating profile in scaled systems. The potential applications for this work are extremely broad and can be adapted to thermochemical reactor configurations using fixed, moving, and fluidized bed modes with nearly arbitrary target axial temperature profiles. We also anticipate system configurations in which the axial temperature profile within a metamaterial can be dynamically modified, which has particular applicability in scenarios where the input power level or feedstock is dynamically varying (*42*, *43*).

## MATERIALS AND METHODS

We show our experimental set up in Fig. 3A, which includes our metamaterial sections packed within a quartz tube and filled with catalyst pellets. The reaction zone is 175 mm long and consists of 14 individual sections of 12.5-mm-long, 38-mm OD cubic SiSiC lattices with strut thicknesses of 1 mm. Two 5-mm-thick 100 PPI SiC disks (ERG Aerospace Corp.) are placed on either end of the susceptor sections to keep the catalyst pellets in place. The catalyst consists of mesoporous gamma phase alumina spherical supports with 18 weight % loading of potassium carbonate (*31*). Each of the lattice sections and SiC disks has a 5-mm hole in the center to accommodate a Hastelloy thermowell (Idaho Laboratories Corp.), which houses a multipoint fiber optic temperature sensor (Technica) that has 15 sensors spaced 15 mm apart. The quartz tube has an inner diameter of 39 mm, is 800 mm long, and is capped on either end with flanges with integrated gas ports and temperature sensor ports. We surround our susceptor with 12.5-mm-thick alumosilicate thermal insulation (Zircar Ceramics, Inc.). Our induction coil consists of nine turns with diameters of 88 mm from 6.35-mm-diameter copper tubing with a spacing of 24.5 mm. The reactor and induction coil are held up with acrylic supports to minimize parasitic coupling and heating from the AC fields.

Each of the 14 SiSiC lattice sections within our reactor has one of two possible electrical conductivities, $\sigma_{\text{high}}$ or $\sigma_{\text{low}}$ depending on the silicon content. Our baseline experiments are conducted with a “uniform” reactor consisting of 14 sections of the $\sigma_{\text{low}}$ lattices, such that the power dissipation profile is consistent with a uniform monolith. We have shown that the two kinds of lattices have identical porosity when packed with the potassium carbonate catalyst, and identical heat transfer properties (see section S2.3). Section S2.1 includes a schematic of the packed uniform reactor, a nonuniform reactor consisting of some low and some high electrical conductivity sections, as well as the axial power dissipation of each of these configurations. The comparison of a uniform reactor to an inversely designed reactor is always conducted at the same flow rate and maintaining the same maximum temperature within the reactor.

Power is supplied to our coil through a signal generator which passes through a power amplifier, matching network, and power meters. A sinusoidal signal is generated at the operating frequency, 3 MHz, and then amplified in a linear amplifier. The signal passes through the first in-line dual directional coupler (Werlatone C3807) before a tunable matching network consisting of two capacitors, one in series with the load (coil and susceptor) and the other in parallel with the load. The purpose of the matching network is to convert the load impedance (the coil and susceptor) from a mostly inductive and slightly resistive load to a purely resistive load with a resistance of 50 Ω. We then use a second in-line directional coupler (Werlatone C3807) after the matching network to measure the power dissipated across the load. Both of the in-line directional couplers are connected to a power meter and its sensors (Agilent N1914A, N8428A), which measure forward, reflected, and net power delivery. We use the power meters to ensure that our load is purely resistive, i.e., the reflected power at the linear amplifier is 0, and measure the net power delivered to the coil and susceptor, which is used when tuning our 1D model.

We use this experimental set up to collect 100 data points consisting of a unique combination of susceptor configuration, flow rate, set outlet temperature, power dissipation, and CO_2_ conversion. We use a custom-developed system to perform closed loop feedback and maintain a desired temperature by adjusting the input power. We control the flow rate using flow controllers (Alicat Scientific) and determine the CO_2_ conversion by analyzing the outlet gasses with a commercial mass spectrometer (MKS Cirrus 3). We use a water trap to condense water out of the outlet gas line before analyzing its composition. Our inlet gas consists of a 3:1:1 ratio of H_2_:CO_2_:Ar, where Ar is used as a reference gas and the CO_2_ conversion is defined as $1-C_{{\text{CO}}_{2}}/C_{\text{Ar}}$ where $C_{i}$ is the measured molar concentration of gas *i*.

## Supplementary Materials

### This PDF file includes:

Supplementary Text Figs. S1 to S13 Tables S1 to S6 References
