# Supplementary material for: Electrified thermochemical reaction engineering with metamaterial reactors
Source: Sci Adv. 2026 Sep 2;12(36):eaeg7217. doi: 10.1126/sciadv.aeg7217 (PMC13537260; doi:10.1126/sciadv.aeg7217)
Supplement: Supplementary file 1 [file sciadv.aeg7217_sm.pdf]

Science Advances
AAAS

Supplementary Materials for

Electrified thermochemical reaction engineering with metamaterial reactors

Ariana B. Höfelmann *et al.*

Corresponding author: Jonathan A. Fan, jonfan@stanford.edu

Sci. Adv. **12**, eaeg7217 (2026)
DOI: 10.1126/sciadv.aeg7217

This PDF file includes:

Supplementary Text
Figs. S1 to S13
Tables S1 to S6
References

S1 Inductively Heated Metamaterial Sections

S1.1 Axial Power Dissipation Trends

We describe an axially non-uniform heating profile for inductively heated susceptors with skin depths below $1.25r_0$ within a uniform magnetic field, which limits the ability to model the power dissipation with a simple analytical model. Here we examine how the axial power dissipation profile varies with skin depth and susceptor length with the assistance of COMSOL Multiphysics modeling. In Fig. S1 we plot the normalized axial power density for different length susceptors at different skin depths. These simulations are performed assuming an electrically conductive, non-magnetic susceptor within an identical, finite coil (the effects of which will be discussed in Section S1.4). A finite coil produces a non-uniform and parabolic magnetic field profile with maximum field strength in the axial center of the coil. It will be shown that the power dissipation of the suceptor is proportional to the square of the magnetic field (Section S1.5). We observe that at small skin depths the axial power profile features higher heating at the ends of the susceptor caused by axial shielding effects (*44*). This effect is characteristic of heating finite susceptors and is well known. However, as it is a function of both size and electrical conductivity, it limits our ability to treat the digital metamaterial sections as independent.

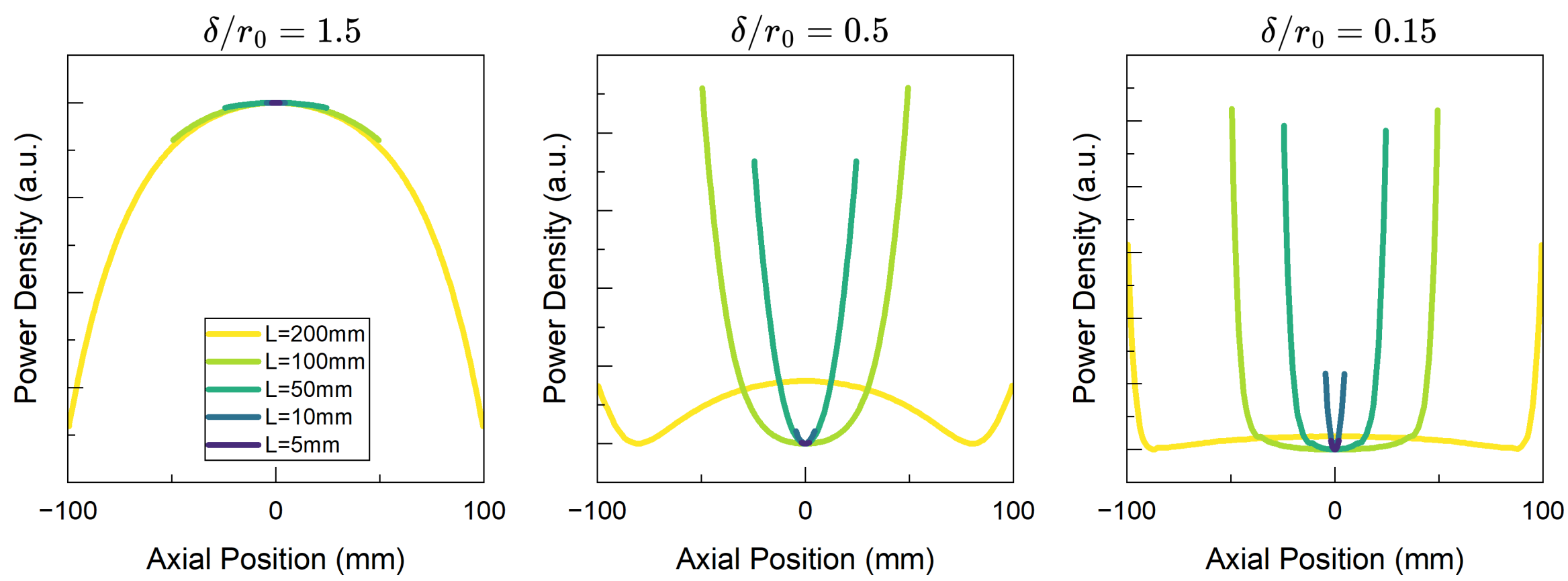

Figure S1: **Axial power dissipation for susceptors of varying conductivity and length**. For susceptors of identical radius and varying length, there is a clear relationship between the skin depth and the axial power density. At comparably high skin depths, shown in the left-most plot, the simulated axial power density follows a roughly parabolic profile, which is directly proportional to the magnitude of the magnetic field squared. As the skin depth increases, such as in the center and right plots, axial shielding effects increase power dissipation at the ends of the susceptor compared to the center, a notable deviation from the magnetic field squared trend.

We further examine the condition at which the axial power profile is no longer dominated by axial shielding effects. We use a metric to assist in determining the skin depth at which axial shielding is negligible, defined as the sum of the square of the difference between the power profile (normalized by maximum) and magnetic field profile (normalized by maximum), $\Sigma(\text{norm}(p(z)) - \text{norm}(B(z)^2))^2$. We find that the transition is a function of skin depth and susceptor length, with a reasonable limit being $\delta/r_0 = 1.25$. In Fig. S2, we plot this metric for multiple susceptor lengths with identical radii

as a function of skin depth, showing that for a reasonable range of susceptor lengths, the metric is close to 0 at skin depths larger than $\delta/r_0 = 1.25$.

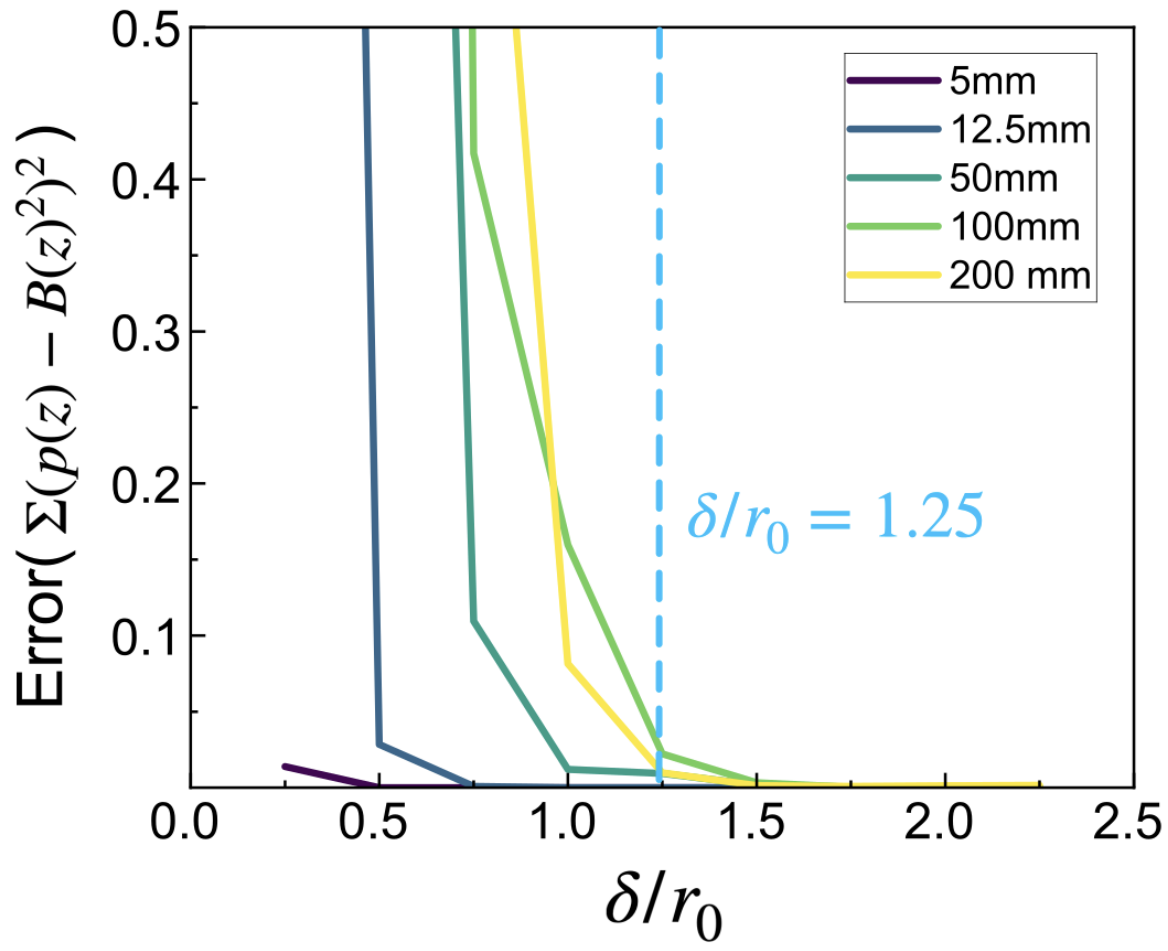

Figure S2: **Error metric for axial Power density and B-field squared**. We plot the calculated error metric for various length susceptors under identical coil conditions and notice the inflection point at which the error metric goes to 0 varies with length. We observe that for all plotted lengths, the error metric goes to 0 by $\sim \delta/r_0$=1.25.

S1.2 Power Dissipation in Susceptor

We claim that analytical expressions for the power dissipated in an inductively heated susceptor can be used to describe the total power dissipated in terms of the coil current, I_{coil}. We further use this property to describe the coupling efficiency. These expressions will also be useful in describing the axial power density. The well-known expression for the volumetric power dissipated in a homogeneous cylinder of length L and radius r_0 is described as: (*45*)

$$P = \iiint \frac{|\vec{J}|^2}{2\sigma_{eff}} dV \tag{3}$$

where $\vec{J}$ is the eddy current density and σ_{eff} is the effective electrical conductivity of the cylinder. Solving Maxwells equations for the current within an infinitely long cylinder provides an expression for the current density in terms of the imposed magnetic field magnitude, B_0, generated by a helical coil placed around the cylinder and driven by an alternating field, and complex wavenumber, $k = \sqrt{-i\omega\mu_0\sigma_{eff}}$ (*46*) :

$$\vec{J}(\rho) = \frac{kB_0}{\mu_0} \frac{J_1(k\rho)}{J_0(kr_0)} \hat{\phi} \tag{4}$$

where J_i is the Bessel function of the ith order, ω is the angular frequency, and μ_0 is the permeability of free space. Using cylindrical coordinates, the above integral becomes:

$$P = \int_0^{2\pi} \int_0^{L} \int_0^{r_0} \frac{1}{2\sigma_{eff}} \left| \frac{kB_0}{\mu_0} \frac{J_1(k\rho)}{J_0(kr_0)} \hat{\phi} \right|^2 \rho d\rho dz d\theta \tag{5}$$

Evaluating the integral provides an expression for the total power dissipation, which can be expressed in terms of the skin depth, $\delta = 1/\sqrt{\mu_0 \pi f \sigma_{eff}}$:

$$P_{diss} = \frac{2\pi\omega B_0^2 L r_0^2}{\mu_0} \text{Im}\left\{ \frac{J_2((1+i)r_0/\delta)}{J_0((1+i)r_0/\delta)} \right\} \tag{6}$$

Using the long solenoid approximation, the imposed magnetic field is $B_0 = \mu_0 N I_{coil}/L_{coil}$, where N is the number of turns and L_{coil} is the coil length. Now, the power dissipation can be written in terms of the coil current:

$$P_{diss} = \frac{2\pi\omega\mu_0 N^2 I_{coil}^2 L r_0^2}{L_{coil}^2} \text{Im}\left\{ \frac{J_2((1+i)r_0/\delta)}{J_0((1+i)r_0/\delta)} \right\} \tag{7}$$

It can be observed that the power dissipation can be written in terms of $I_{coil}^2 R_{sus}$ by pulling out the current term and describing the remaining terms as the AC resistance of the susceptor, R_{sus}:

$$R_{sus} = \frac{2\pi\omega\mu_0 N^2 L r_0^2}{L_{coil}^2} \text{Im}\left\{ \frac{J_2((1+i)r_0/\delta)}{J_0((1+i)r_0/\delta)} \right\} \tag{8}$$

This expression is valid for both high aspect ratio (i.e. long) susceptors as well as the truncated metamaterial sections used. The coil AC resistance can be described using the improved Dowell's

method which factors in skin and proximity effects (*47*):

$$R_{coil} = \frac{\pi^{3/4} D_{coil} N^{3/2}}{\sigma_{coil} \delta_{coil} \sqrt{r_{coil} L_{coil}}} \tag{9}$$

where D_{coil} is the coil diameter, σ_{coil} is the electrical conductivity of the coil, δ_{coil} is the skin depth of the coil, r_{coil} is the radius of the coil tube.

The coupling efficiency is defined as the relative power dissipated in the susceptor compared to the whole system (susceptor and coil). It can also be described by the AC resistance of the susceptor and coil using the property that the power dissipated in each can be described as $P = I_{coil}^2 R$:

$$\eta_{coupling} = \frac{P_{sus}}{P_{sus} + P_{coil}} = \frac{I_{coil}^2 R_{sus}}{I_{coil}^2 R_{sus} + I_{coil}^2 R_{coil}} = \frac{R_{sus}}{R_{sus} + R_{coil}} \tag{10}$$

As we note, the AC resistance expressions holds for susceptors of various lengths, including our truncated sections. The total AC resistance of our susceptor consisting of 14 sections will be the sum of the resistance of each of the sections.

S1.3 Experimental AC Resistance of Metamaterial Sections and Full Susceptors

We experimentally characterize the AC resistance of full length susceptors, consisting of 14 sections of SiSiC metamaterial lattices, induction coil, and individual sections to determine the effective electrical conductivity, AC resistance ratio, and coupling efficiency. To measure the AC resistance we take small-signal impedance measurements with a vector network analyzer (Bode 100) of a lattice section placed within a helical coil. The vector network analyzer measures impedance parameters; the real part of impedance provides AC resistance. We extract the AC resistance of the susceptor by first taking an impedance measurement of the coil with the susceptor placed within it and then a second measurement of just the coil. The difference of these two measurements is the AC resistance of the susceptor.

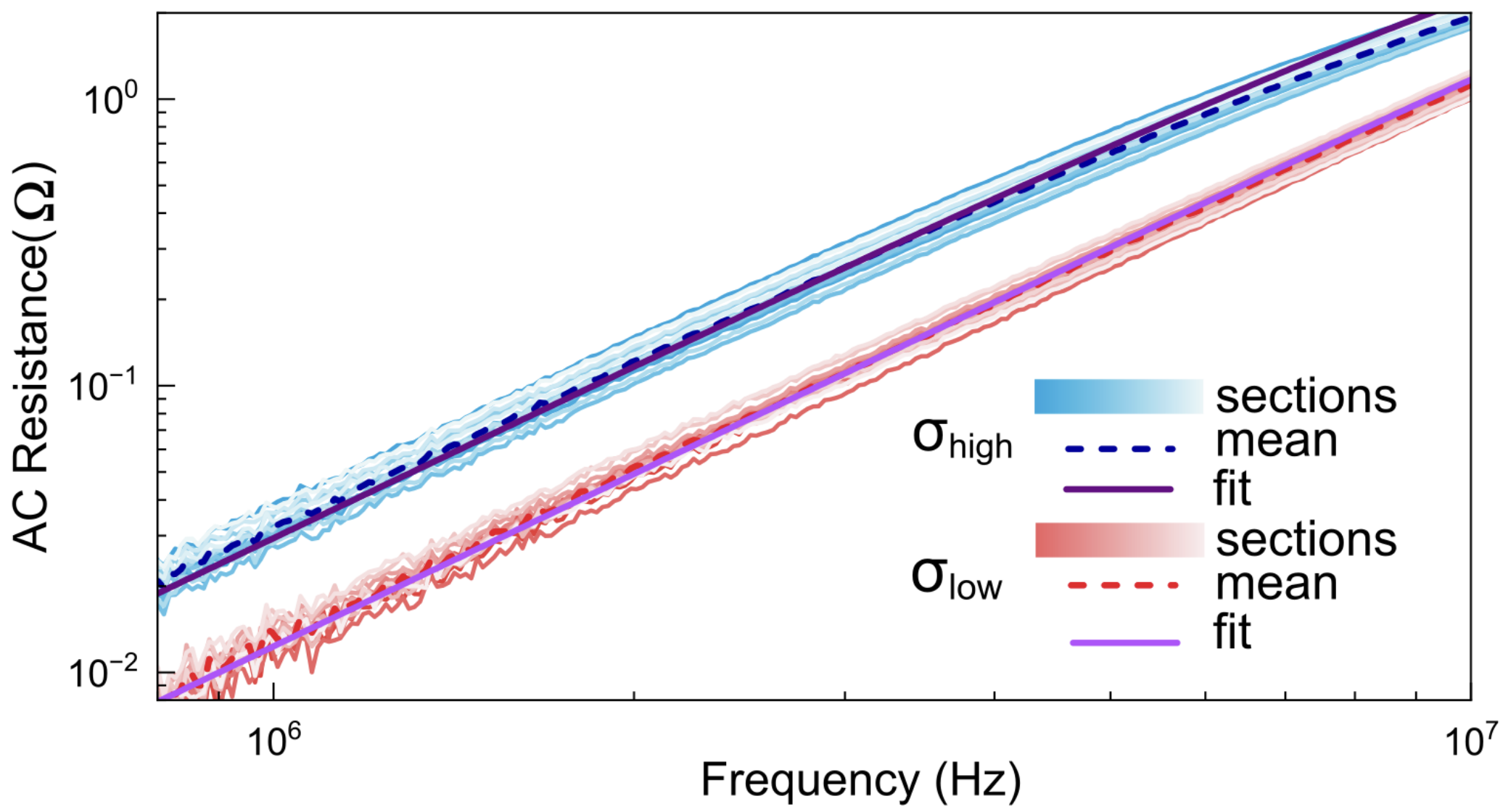

Figure S3: **Experimental AC resistance measurements and fit to analytical model**. The experimental AC Resistance as function of frequency for each of the high conductivity (blue) and low conductivity (red) metamaterial sections. The average of each group of sections (dashed lines) was used in extracting the electrical conductivity by fitting analytical models for AC resistance (solid lines).

The two kinds of SiSiC lattices used have identical geometries and different silicon loadings, which result in different electrical conductivities. The 12.5 mm lattice sections were created by cutting a 200 mm long lattice into 14 pieces via EDM. Fig. S3 shows the AC resistance of each section of the two kinds of lattices as well as the average AC resistance. Variations in resistance are caused by broken struts and variations in section length due to manufacturing error. The effective electrical conductivity was extracted by fitting equation 8 using a least squares method. We determined the electrical conductivity of the lattices were 115 S/m and 48 S/m respectively.

S1.4 Finte Coil Effects

When alternating current is driven through a finite helical coil, an alternating magnetic field is generated with maximum magnitude in the axial center of the coil and reduced magnitude at the ends. An analytical expression for the magnitude of the magnetic field, $|\vec{B(z)}|$ as a function of axial position can be derived. We will consider a coil of length L_{coil} and radius R_{coil}, with current I passing through. The Biot-Savart Law defines the magnetic field due to current passing through a wire as:

$$\vec{B_0} = \frac{\mu_0}{4\pi} \int \frac{I\vec{dl} \times \hat{r}}{r^2} \tag{11}$$

where μ_0 is the magnetic permeability of free space, I is the current magnitude in a wire of length $\vec{dl}$, over which the integral is evaluated, and r is the distance to the point at which the field is being computed. Equivalently, the differential magnetic field can be described as:

$$d\vec{B_0} = \frac{\mu_0}{4\pi} \frac{I\vec{dl} \times \hat{r}}{r^2} \tag{12}$$

The magnetic field in the radial center of a coil with the above dimensions is:

$$d\vec{B_0} = \frac{\mu_0 R^2 dI}{2(z^2 + R_{coil}^2)^{3/2}} \hat{z} \tag{13}$$

where z is the axial coordinate, with 0 at the center of the coil. The differential current can be written in terms of the axial coordinate, $dI = (NI/L)dz$, where N is the number of loops in the coil, and I is the magnitude of current in the coil. Substituting for dI, it is then possible to solve the above integral for the total magnetic field as a function of z:

$$\vec{B_0} = \frac{\mu_0 NI}{L_{\text{coil}}} \left(\frac{(z + L_{\text{coil}}/2)}{\sqrt{(z + L_{\text{coil}}/2)^2 + R_{coil}^2}} + \frac{(L_{\text{coil}}/2 - z)}{\sqrt{(L_{\text{coil}}/2 - z)^2 + R_{coil}^2}} \right) \hat{z} \tag{14}$$

With this description, the magnitude of the magnetic field within the radial center of the coil can be described along the length of the coil.

S1.5 Axial Power Profile

The axial power dissipation, $\alpha(z)$, is defined as the power density along the center of the reactor, in units of W/m^3. It is necessary to describe the axial power density as only a function of axial position and reactor configuration, i.e. the electrical conductivity, the operating frequency, and coil geometry. It is assumed that each of the 14 sections which make up the reactor have uniform, and known, electrical conductivity. An expression for axial power density can be derived using the above expressions for the total power dissipated in a susceptor. Recall the power dissipation in a susceptor placed in an infinite coil with uniform magnetic field:

$$P_{\text{infinite}} = \int_0^{2\pi} \int_0^L \int_0^{r_0} \frac{1}{2\sigma_{eff}} \left| \frac{kB_0}{\mu_0} \frac{J_1(k\rho)}{J_0(kr_0)} \hat{\phi} \right|^2 \rho d\rho dz d\theta \tag{15}$$

For a finite coil, the imposed magnetic field, B_0 can be described as a function of z as shown in equation 14. Additionally, the above volumetric integral can be split up into the product of three independent integrals.

$$P_{\text{finite}} = \frac{1}{2\sigma_{\text{eff}}} \int_0^{2\pi} d\theta \int_0^L \frac{B_0(z)^2}{\mu_0^2} dz \int_0^{r_0} \left| \frac{k^2 J_1(k\rho)}{J_0(kr_0)} \hat{\phi} \right|^2 \rho d\rho \tag{16}$$

Where the θ-dependent integral will evaluate to 2π since the system is axis-symmetric, the z-dependent integral is only dependent on the magnetic field in the coil, and the ρ-dependent integral is proportional to the measured AC resistance of the susceptor and determines the features of the AC resistance curve (such as the rollover point). The goal is to describe the axial power density as a function of axial position only. The above integral can be described as in terms of some constant, which will depend on AC resistance, the cross-sectional area, which arises when the integral is evaluated, and the integral term in the z-direction:

$$P_{\text{finite}} = C \times \text{area} \int_0^L B_0(z)^2 dz \tag{17}$$

The constant, as mentioned above is proportional to the AC resistance and therefore a function of frequency. The constant can be moved into the integral:

$$P_{\text{finite}} = \text{area} \times \int_0^L CB_0(z)^2 dz \tag{18}$$

This is the total power dissipated in a uniform susceptor of length L within a finite coil with a uniform conductivity. The axial power density, in units of W/m^3 is described as:

$$\alpha(z) = CB(z)^2 \tag{19}$$

This description of the power density ensures that total power is conserved. For example, if 100 W is dissipated in the susceptor, then C should be specified such that $area \times \int_0^L CB_0(z)^2 dz$ is equal to 100 W, or that $C = 100/(\text{area} \times \int_0^L B_0(z)^2 dz)$.

For a non-uniform susceptor, the constant C now becomes a function of axial position, and is determined by the AC resistance of the the susceptor sections and the total power dissipated. Consider the axial power dissipation of a non-uniform susceptor in a finite coil consisting of two

adjacent sections with conductivities σ_{high} and σ_{low}, identical to our metamaterial susceptor. The length of the low conductivity section is L_1 and the length of the high conductivity section is $L_2 = L - L_1$.

The total power dissipated in the susceptor is the sum of the power dissipated in each of the 2 sections, which can each then be described using equation 18:

$$P = P_1 + P_2 \tag{20}$$

$$P = \text{area} \times \int_0^{L_1} C_1 B_0(z) dz + \text{area} \times \int_{L_1}^{L} C_2 B_0(z) dz \tag{21}$$

Where C_1 and C_2 are appropriate constants depending on the conductivity of each of the sections. We will now define $C(z)$ as a piecewise function of axial position, $C(z) = C_R(z)S$, the product of a piecewise function C_R and a scaling factor S. The AC Resistance Ratio, defined as $R_{\text{high}}/R_{\text{low}}$ and be used to described the piecewise function $C_R(z)$:

$$C_R(z) = \begin{cases} 1, & \text{if } z \leq L_1 \\ R_{\text{high}}/R_{\text{low}}, & \text{if } L_1 < z \leq L \end{cases} \tag{22}$$

The above expression now becomes:

$$P = \text{area} \times \int_0^{L} S \times C_R(z) B_0(z)^2 dz \tag{23}$$

where S is a constant used to preserve the total power dissipated in the susceptor. S can generally be defined as:

$$S = \frac{P}{\int_0^L C_R(z) B_0(z)^2 dz} \tag{24}$$

where P is the total power dissipated in the susceptor. With this definition of S and $C_R(z)$ the axial power dissipation for a non-uniform susceptor is fully defined as $\alpha(z) = SC_R(z)B_0(z)^2$. This description can be used to describe the power dissipated in a susceptor of arbitrary length and number of sections with known AC resistance.

The analytical solution for the axial power presented here was verified using COMSOL simulations and plotting in Fig. S4. The normalized axial power density was plotted for three different susceptor configurations, showing good agreement between the analytical solution and simulation.

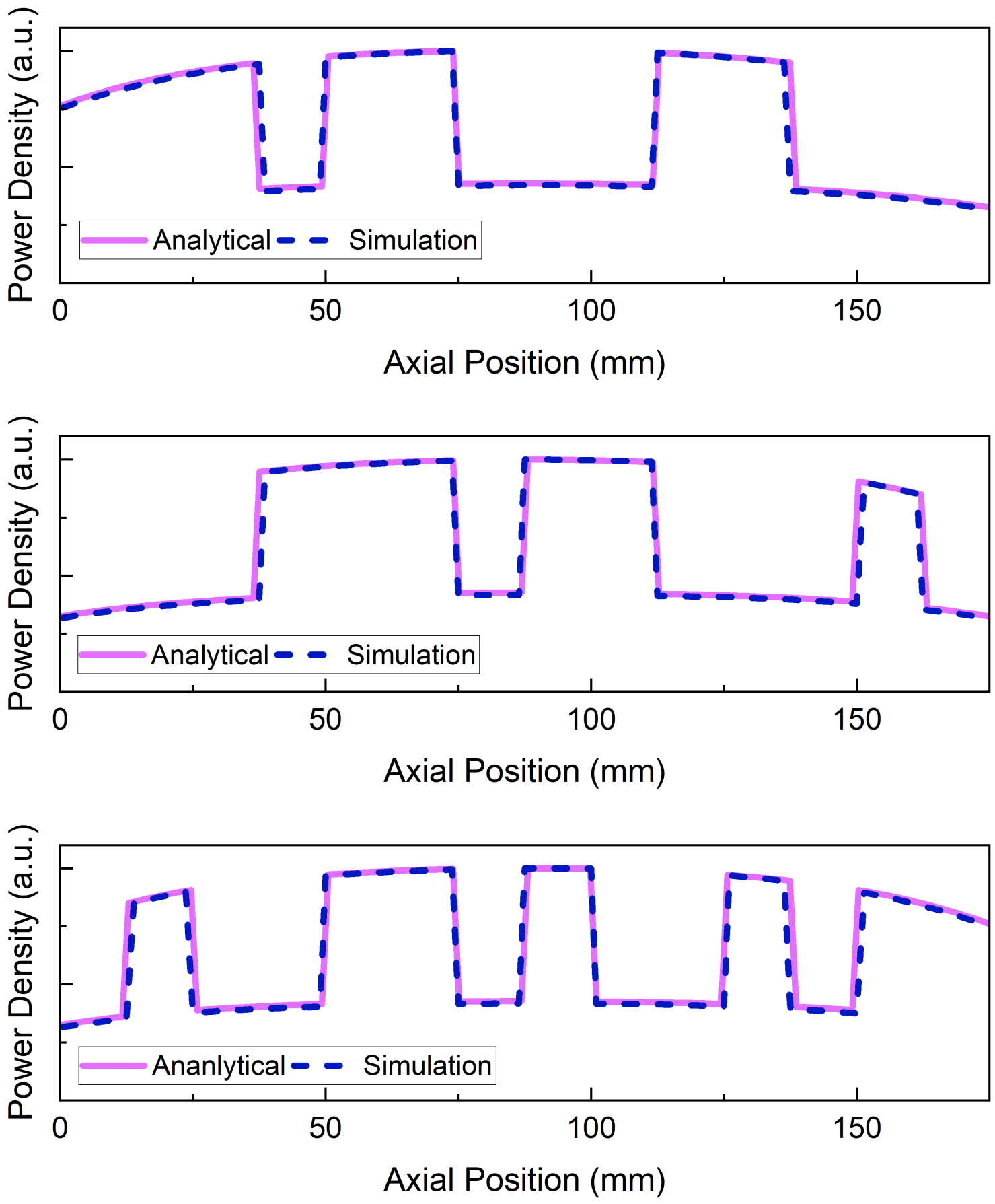

Figure S4: **Comparison of axial power density, analytical vs. simulation**. The axial power density was simulated for three different susceptor configurations using identical coil and susceptor geometries. Comparison of the simulated power profiles to the analytical solution presented here showed minimal deviation.

S1.6 Discrete vs. Continuous Susceptors

We claim that our discrete metamaterial reactor scheme can exhibit the same level of temperature control as continuous or multi-valued schemes. To verify this claim, we perform multiphysics simulations using COMOSL as well as our 1D modeling scheme. The axial temperature profiles for different periodic and aperiodic arrangements of susceptor sections are presented in Fig. S5 and display very similar temperature profiles for both digital and continuous susceptor variants.

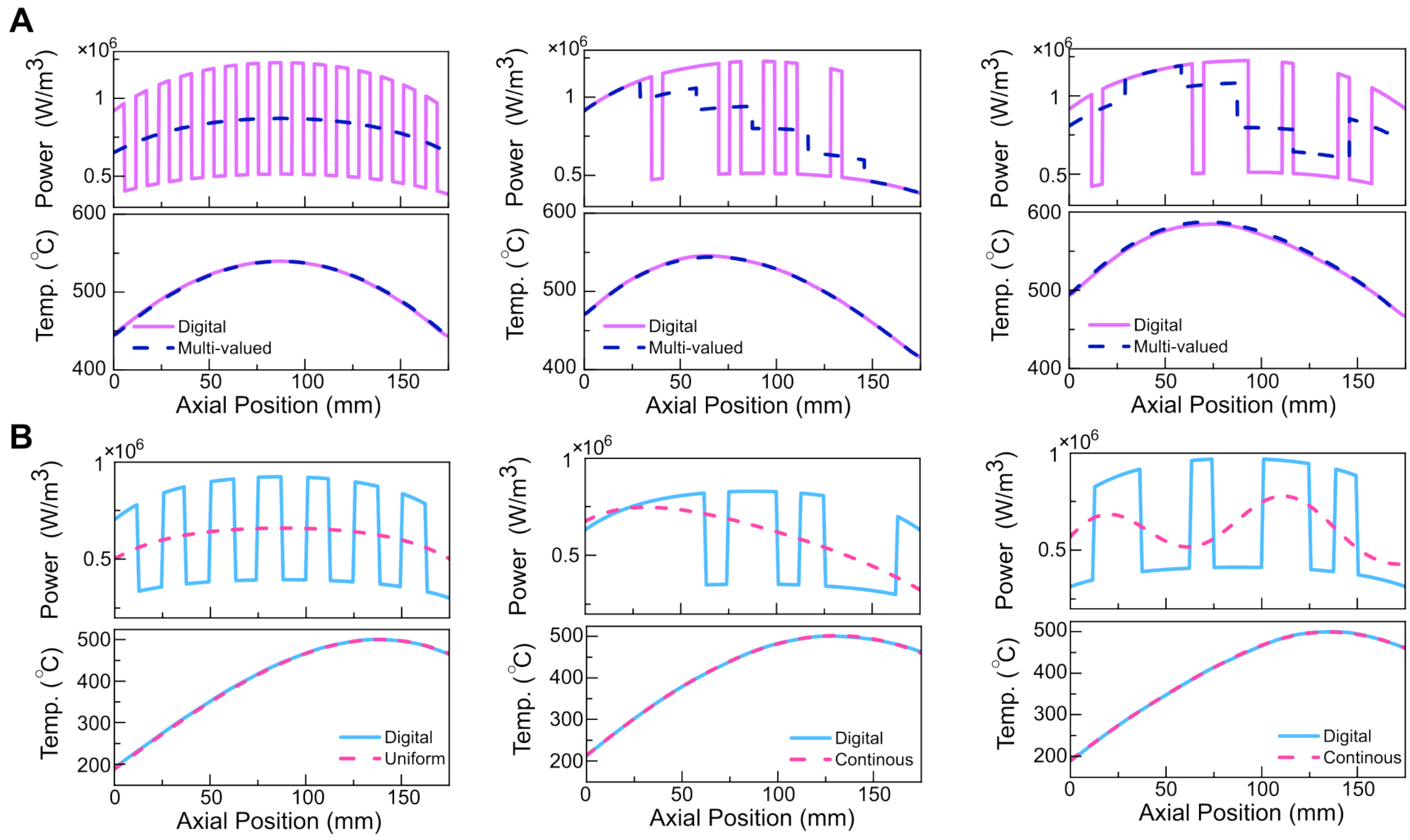

Figure S5: **Power density and temperature profile of discrete, multi-valued, and continuous susceptors**. In (**A**), we plot the axial power density (top row) for periodic and aperiodic discrete susceptors with minimum section lengths of 5 mm as well as equivalent multi-valued structures where subsections of digial susceptors are grouped to match the power dissipated in a single-valued section. The axial temperature profile (bottom row) was simulated assuming only conduction losses. (**B**) plot of the axial power density (top row) of periodic and aperiodic digital susceptors and equivalent susceptors with continuous electrical conductivity and power densities. The axial temperature profile (bottom row) is plotted assuming a flow rate of 4 slpm and a maximum temperature of 500°C.

S2 Experimental Set Up

S2.1 Experimental Set-up and Data Collection

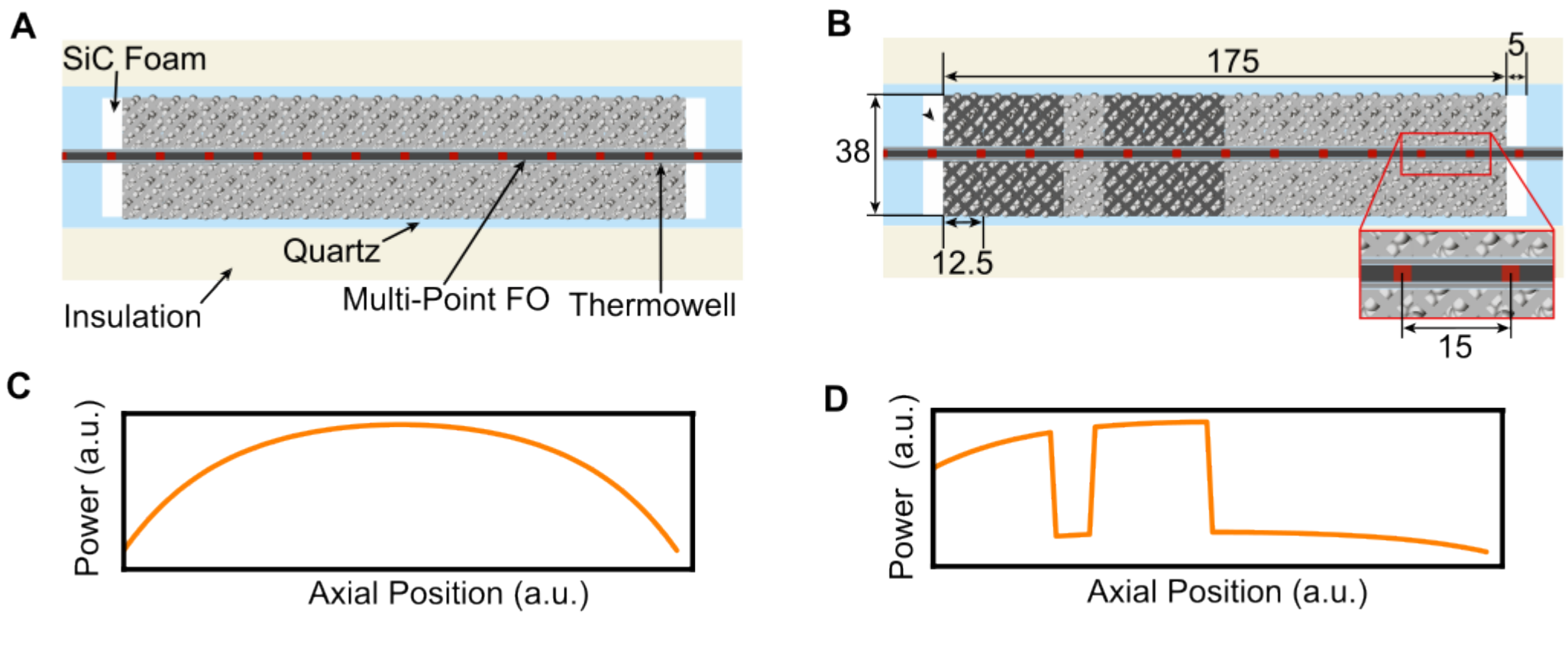

Figure S6: **Uniform and inverse designed reactor experimental set up and axial power dissipation**. (**A-B**) Schematic of the experimental set up for a uniform (left) reactor consisting of 14 sections of catalyst-packed low electrical conductivity lattices and a non-uniform (right) reactor consisting of 14 sections of both high (dark grey) and low (light grey) electical conductivity lattices. (**C-D**) Plots of the axial power dissipation for our uniform, i.e. baseline, (left) reactor and (ii) a non-uniform (right) reactor configuration.

As described in the main text, our benchmark reactor configuration consists of 14 sections of the low electrical conductivity lattice packed with catalyst pellets, which has identical pressure drop and thermal properties to the configurations consisting of both kinds of lattices. Labeled schematics of the uniform benchmark and a single non-uniform reactor configuration, as well as their respective axial power profiles, can be found in Fig. S6.

S2.2 Resolution of Axial Temperature Measurements

We use a resolution of 15 mm when taking axial temperature measurements, totaling 13 points along our 175 mm packed bed. To confirm that there are no local temperature fluctuations, we took 5 mm resolution measurements using our multi-point fiber optic temperature sensor on a motorized stand. When conducting the measurement we held the input power steady to minimize transient temperature fluctuations and held the sensor at each position for two minutes to ensure the sensor had reached thermal equilibrium with the surroundings. We used a non-uniform reactor at a flow rate of 2500 h^{-1} GHSV with a maximum temperature of 500°C and, as seen in Fig. S7, observe a smooth profile which agrees with our simulations and does not have any features to suggest temperature fluctuations within an individual section, despite the discrete nature of our structure. Therefore, we conclude that the interpolation of a 15 mm resolution measurement is sufficient for our purposes.

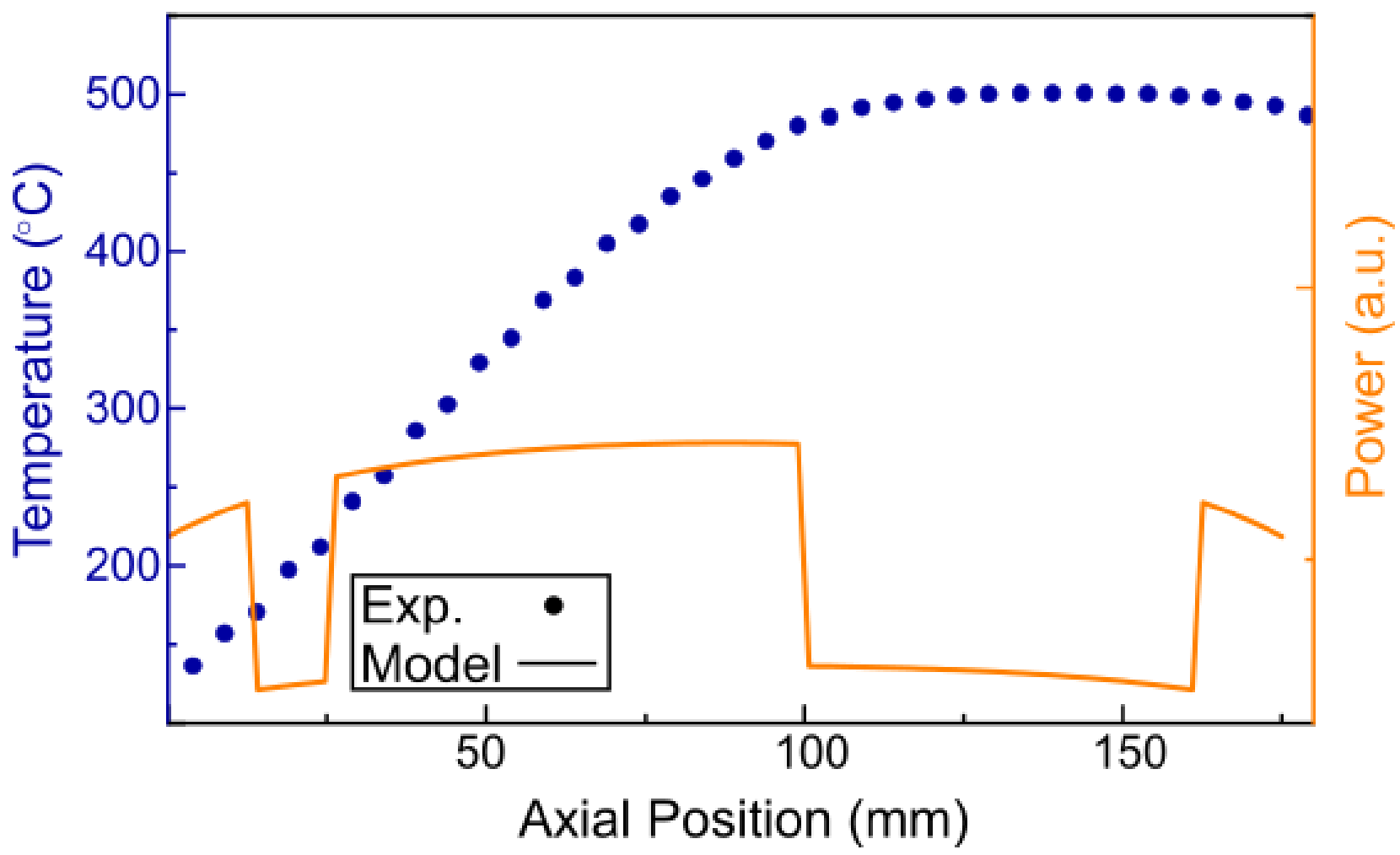

Figure S7: **High resolution temperature measurements**. Plotted is experimental axial temperature measurements taken with a resolution of 5 mm (blue dots) as well as the axial power dissipation of this structure (orange line). Despite discrete changes in the electrical conductivity, i.e. higher or lower power dissipation, the temperature profile remains smooth.

S2.3 Flow Regime

The 1D model implemented assumes negligible pressure drop and mininal shear effects, such that axial changes in flow can be neglected and flow is fully laminar. Additionally, we assume that the effective porosity is identical for each of the two SiSiC lattice types when packed. We verify these assumptions by taking pressure measurements using a differential pressure gauge (Omega PX419-005DWU5V) of each of the packed lattice sections. The 14 of each kind of lattice sections were packed within a 39 mm inner diameter quartz tube, filled with potassium carbonate catalyst pellets, and held in place with SiC disks. We take additional measurements to account for pressure drop in the empty tube and the SiC disks. All pressure drop measurements are taken at room temperature while flowing nitrogen gas. The pressure drop was fit using the Ergun equation and a least squares method to determine the fitting coefficients (*48*):

$$\frac{\Delta P}{L} = A\frac{(1-\epsilon)^2\mu}{\epsilon^3 d_p^2}u + B\frac{(1-\epsilon)\rho}{\epsilon^3 d_p}u^2 \tag{25}$$

where ΔP is the pressure drop, L is the bed length, a and b are fitting coefficients, μ is the dynamic viscosity of the gas, u is the superficial velocity off the gas, d_p is the particle diameter, and ρ is the density of the gas. Fig S8 shows the experimental pressure drop and fit for each of the two lattices, showing near identical pressure drop as a function of flow rate.

To characterize the operating flow regime, we plot the friction factor vs. the pore-averaged Reynolds number, Fig. S8. We use a porosity of $\epsilon = 0.3$ for both of the lattices, which was determined from a measurement of the volume fraction of the catalyst packed bed, assuming 135 g of catalyst used and an ordered packed bed ($\epsilon_{cat} = 0.26$) (*49*), and the volume fraction of the SiSiC lattice, which was designed to be $\epsilon_{lat} = 0.92$. We find the packed lattices to have an effective porosity of ~ 0.3. The friction factor, f and pore-averaged Reynolds number are defined as (*50*):

$$f = \frac{\Delta P d_p \epsilon^3}{L\rho u^2(1-\epsilon)} \qquad \mathrm{Re} = \frac{\rho u d_p}{\mu(1-\epsilon)} \tag{26}$$

A pore-averaged Reynolds number below 10 indicates fully laminar flow (*51*). We determine that at our operating conditions the maximum Reynolds number is ~ 7, and therefore we are in a fully laminar regime.

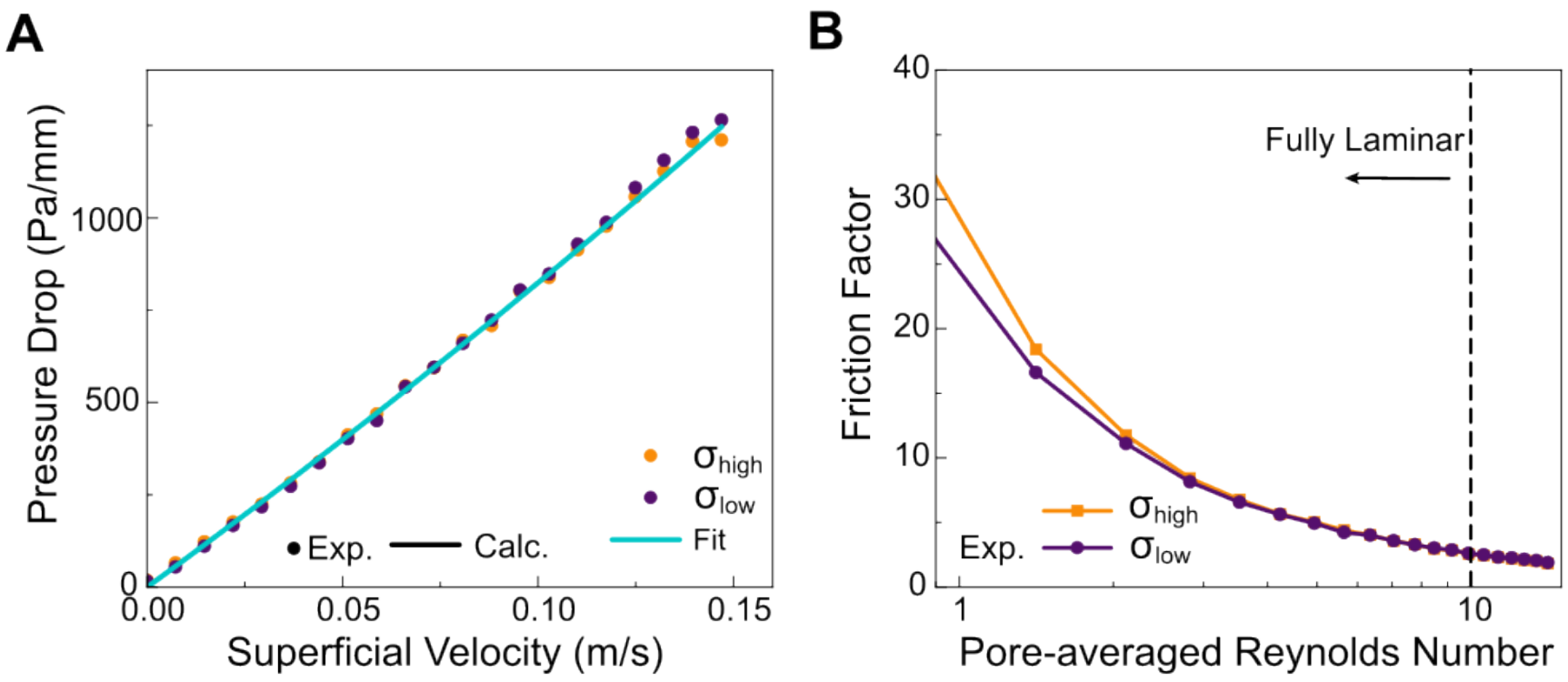

Figure S8: **Pressure drop and friction factor of packed high electrical conductivity and low electrical conductivity lattices.** (**A**) We plot the pressure drop divided by bed length for a bed of high and low electrical conductivity lattices packed with potassium carbonate catalyst pellets as well as the Ergun equation with fit coefficients of A=24 and B=0.17 ($R^2 = 0.99$). (**B**) We plot the friction factor vs. Reynolds number for each of the packed lattice types.

S3 Model Construction and Validation

S3.1 1D Model

We describe the temperature within the metamaterial reactor can be described using a coupled mass balance and energy balance.

Energy Balance
The 1D pseudo-homogeneous energy balance is

$$k_{eff}\frac{d^2T}{dz^2} - \rho c_p \epsilon u \frac{dT}{dz} - \eta r(T,\vec{C})H_{rx}(T) + \alpha(z) = 0 \tag{27}$$

where k_{eff} is the thermal conductivity, which we assume is inversely proportional with temperature, $k_{eff} = C/T$ where C is some scaling factor (*52*) (see following discussion on radiative effects), ρ is the density of the gas moving through the reactor, c_p is the heat capacity of the gas moving through the reactor, ϵ is the effective porosity of the packed susceptor, u is the gas superficial velocity, η is the catalyst effectiveness factor, $r(T,\vec{C})$ is the reaction rate, $H_{rx}(T)$ is the heat of reaction, $\vec{C}$ is a vector of molar concentrations where C_i is the molar concentration of species i, and ν_i is the stoichiometric coefficient of species i (*53*) (*39*) (*40*). $\alpha(z)$ is the axial power density from digital metamaterial susceptor heating. Convective boundary conditions are applied at the inlet and outlet of the reactor defined as:

$$h(T - T_\infty) = -k_{eff}\frac{dT}{dz} \tag{28}$$

where h is the heat transfer coefficient.

Mass Balance
The mass balance described is derived from the conversation of mass in a reactor section dz where the reaction is occurring assuming plug flow operation. 1D volume mass balance for reacting species i:

$$F_i(z) - F_i(z+dz) + \text{area} \times \nu_i r_i(\vec{C},T)dz = 0 \tag{29}$$

where F_i is the molar flow rate, area is the cross-sectional area, and C_i is the molar concentration of species i. Using the fact that the molar flow rate can be expressed in terms of the superficial velocity, $F_i = \text{area} \times u(T)C_i(T)$, and that the superficial velocity scales with temperature, $u = u_{\text{STP}} \times T/300$, the mass balance becomes:

$$\frac{C_i}{T}\frac{\delta T}{\delta z} + \frac{\delta C_i}{\delta z} = \frac{\nu_i r_i(\vec{C},T)}{u(T)} \tag{30}$$

Reaction Rate
The chemical equation for RWGS is:

$$\text{CO}_2 + \text{H}_2 \leftrightarrow \text{CO} + \text{H}_2\text{O} \tag{31}$$

The reaction rate can be expressed using elementary kinetics:

$$r_{\text{forward}} = C_{\text{CO}_2}C_{\text{H}_2}A_f \exp\left(\frac{-E_f}{R_{gas}T}\right) \tag{32}$$

$$r_{\text{reverse}} = -C_{\text{CO}}C_{\text{H}_2\text{O}}A_r \exp\left(\frac{-E_r}{R_{gas}T}\right) \tag{33}$$

where A_f and A_r are the pre-exponential factors of the forward and reverse reactions, respectively, and E_f and E_r are the forward and reverse activation energies, respectively. The total reaction rate $r(C_i, T_i)$ can be defined as:

$$r = r_{\text{forward}} + r_{\text{reverse}} \tag{34}$$

The rate parameters, A_f, E_f, A_r, and E_r relate to each other at equilibrium in the following manner, due to the fact that the forward and reverse reactions are equal.

$$r_f = -r_r \rightarrow \frac{A_f}{A_r}\frac{1}{K}\exp\left(\frac{E_r - E_f}{R_{gas}T}\right) = \frac{A_f}{A_r}\exp\left(\frac{\Delta G^\circ(T)}{R_{gas}T}\right)\exp\left(\frac{E_r - E_f}{R_{gas}T}\right) = 1 \tag{35}$$

where K is the equilibrium constant and $\Delta G^\circ(T)$ is the Gibbs free energy. We observe that ΔG° is approximately linear with temperature and use this fact to relate the activation energies and pre-exponential factors to each other

$$E_r - E_f = -\Delta G^\circ \tag{36}$$

$$A_r = A_f \exp\left(\frac{d\Delta G^\circ/dT}{R_{gas}}\right) \tag{37}$$

Therefore, in describing the reaction kinetics we need to only fit two parameters, A_f and E_f.

S3.2 Radial Temperature Gradients

In constructing the 1D pseudo-homogeneous heat transfer and mass balance model, we assume that radial temperature gradients are negligible. We verify this assumption through 2D multiphysics simulations. These simulations are performed in COMOSL and couples inductive heating, flow through porous media, and reaction kinetics. In Fig. S9 we plot the simulation environment, as well as the axial and radial temperature profiles of a uniform susceptor. We observe good agreement of the axial temperature profile (taken at the radial center of the reactor) between the experimental and simulation results at identical flow rates and maximum temperatures. We also observe that the radial temeprature gradient within the susceptor is negligible, due to the high thermal conductivity of the SiSiC lattices used as well as the volumetric nature of inductive heating. Therefore, we do not need to explicitly account for radial heat loss within our model and can assume that there is negligible, if any, net radial heat flux within the center of the reactor.

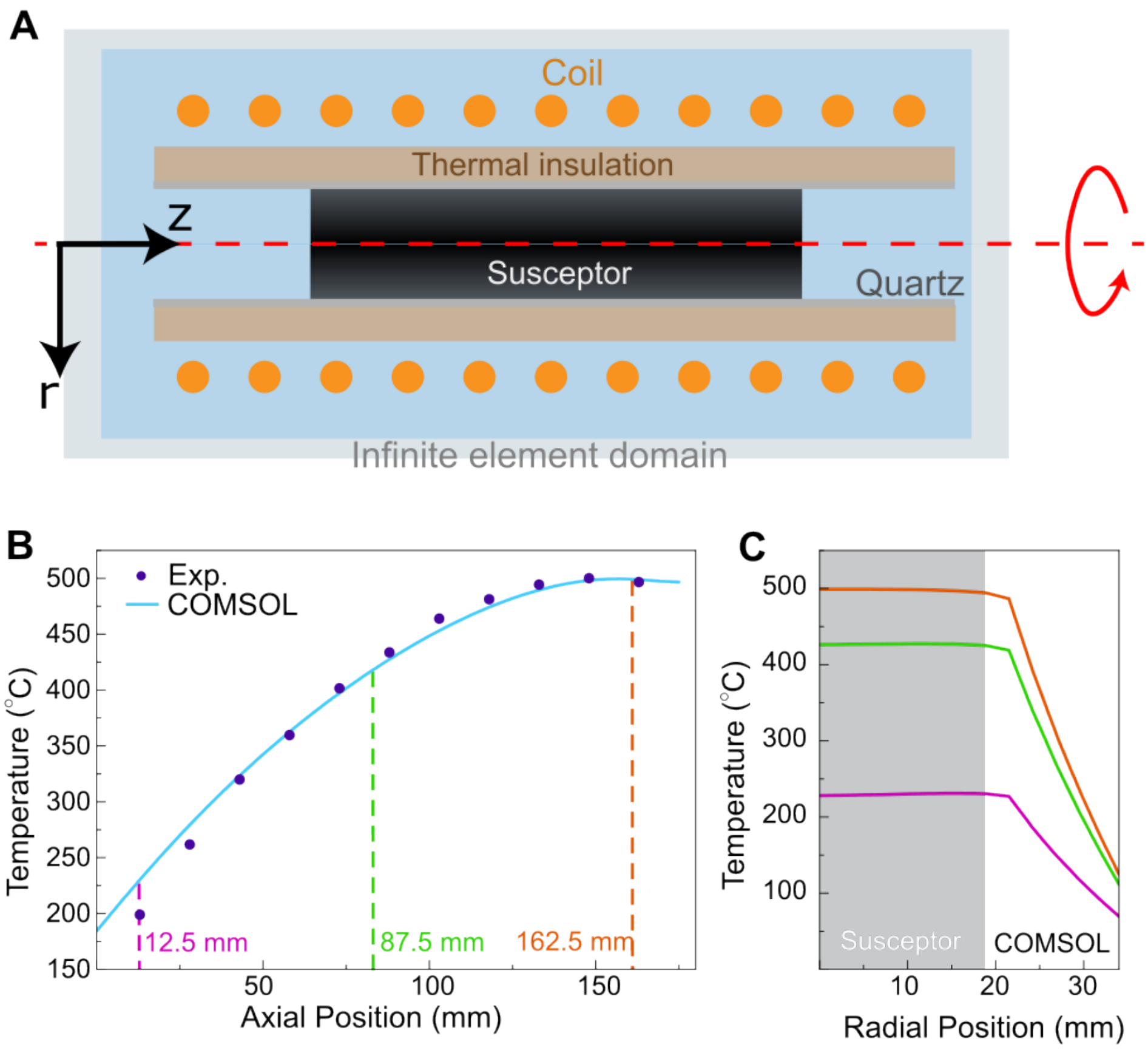

Figure S9: **2D heat transfer simulation.** (**A**) Simulation set up of inductively heated reactor. (**B**) Axial temperature profile predicted with COMSOL model compared to experimental data for a uniform susceptor with a maximum temperature of 500 °C and 2500 (h^{-1}) GHSV. Vertical lines denote locations of radial temperature curves. (**C**) Radial temperature gradients through susceptor, quartz, and insulation at 12.5 mm, 87.5 mm, and 162.5 mm from the reactor inlet.

We can further verify the temperature uniformity by evaluating the radial Biot number. A Biot number less than 1 represents a system with negligible temperature gradients. The Biot number is defined as

$$\mathrm{Bi} = \frac{hL_c}{k} \tag{38}$$

where h is the heat transfer coefficient from the surface of the solid body (i.e. the susceptor), L_c is the characteristic length, and k is the thermal conductivity. The characteristic length is the radius of the suceptor, 0.019 m, and the thermal conductivity is $\approx$ 17.5 W/mK (average for the temperatures considered here, fit from experimental data collected as described in Section S3.3). The effective heat transfer coefficient is calculated from the thermal resistance through the quartz wall and insulation.

$$R_{th} = R_{quartz} + R_{insulation} \tag{39}$$

Assuming the experimental set up described in Section S2.1, and a thermal conductivity of 0.1 W/mK for the insulation (*54*)and 1.39 W/mK for quartz (*55*). The radial Biot number is then 5×10^{-4}. As this is much smaller than 1, it is reasonable to assume that there is no significant radial temperature gradient.

S3.3 Parameter Extraction

We perform parameter extraction through a least squares method which fits each of the parameters in a series of steps by sweeping possible values.

Table S1: Parameters in 1D Model and extraction methods

Parameter	Expression	Fitting Method
k_{eff}	$= C/T$	least squares fit: loss = $(T_{exp} - T_{predict})^2$
ϵ	=0.3	measured experimentally (SI 2.2)
ρ	$= PM/(R_{gas}T)$	Ideal gas law
c_p	=f(T)	known relations with T (*56*)
u	$u_{\mathrm{STP}}/300 \times T$	from set flow rate
η	=1	assume reaction rate is ideal
$H_{rx}(T)$	$= H^{\circ}_{rx} - H(T)_{reac.} + H(T)_{prod.}$	known relations for H (*56*)
A_f,E_f	Section S3.1	least squares fit: loss = $(\%CO_{2,exp} - \%CO_{2,predict})^2$
$\alpha(x)$	Section S1.5	analytical solution and exp. power
$h_{in}(u,T), h_{out}(u,T)$	A(u)exp(B(u)(T-C(u)))	least squares fit: loss = $(T_{exp} - T_{predict})^2$

We extract the effective kinetics parameters, the pre-exponential factors and activation energies using the mass balance equation and experimental temperature values and use a least squares method where the loss is the predicted compared to the experimental conversion values. We then set the effective kinetics parameters and fit the effective thermal conductivity and boundary conditions using a least squares method where the loss is the predicted vs actual temperature profile. The effective thermal conductivity used is the average of the best fit conductivities for each of the training points. With a set thermal conductivity, the inlet and then outlet boundary conditions are extracted for each of the training points and fit to an exponential function. A summary of parameters and extraction methods can be found in Table S1.

As discussed above, we assume that the radiative contribution to the effective thermal conductivity of the bed is negligible in constructing our model. We can further estimate the radiative contribution using the Rosseland effective radiative conductivity approximation for optically thick porous media, $k_{\mathrm{rad}} = 16\sigma T^3/(3\beta)$, where σ is the Stefan–Boltzmann constant, T is the absolute temperature, and β is the Rosseland mean extinction coefficient, which characterizes the attenuation of thermal radiation within the porous structure due to absorption and scattering (*57*) (*58*). Physically, a larger β corresponds to a shorter photon mean free path and therefore weaker radiative heat transfer. For a homogeneous porous medium, β can be approximated as $\beta \approx 8(1 - \varepsilon)/d_m$, where ε is the porosity and d_m is the characteristic radiative length scale of the porous medium. In our packed bed reactor, the radiative transport length is not governed by the ~1 mm catalyst particles occupying the open volume. Therefore, $d_m \approx 1$ mm represents a conservative upper-bound estimate for the effective radiative length scale, while the actual value is expected to be even smaller due to additional obstruction and scattering introduced by the packed particles. Using $\varepsilon = 0.90$, $d_m = 1$ mm, and $T = 823$ K (550 °C), we obtain $\beta \approx 800$ m^{-1}, giving $k_{\mathrm{rad}} \approx 0.21$ W/(mK). This value is relatively small and negligible compared to the effective conductive heat transfer captured by our model and experiment.

After performing the described fitting process, we find the effective thermal conductivity to be $10713/T$ which results in thermal conductivities between 12-22 W/(mK) at the temperatures we consider, much larger than the contribution of radiative heat transfer. We additionally find that the forward activation energy E_f, is 150 kJ/mol, and the forward pre-exponential factor, A_f, is

9.7×10^9. The coefficients for the inlet and outlet boundary conditions, whose expression can be found in Table S1, are shown in Table S2 and Table S3, respectively. We specify the boundary conditions as both a function of flow rate (GHSV) and inlet/outlet temperature.

Table S2: Coefficients of the inlet boundary condition (h_{in})

GHSV (h^{-1})	A(u)	B(u)	C(u)
500	154	-0.0016	599
1000	134.13	-0.0027	593
1500	97	-0.0036	616
2000	80	-0.0047	576
2500	69	-0.0051	589

Table S3: Coefficients of the outlet boundary condition (h_{out})

GHSV (h^{-1})	A(u)	B(u)	C(u)
500	144	0.0048	540
1000	102	0.0019	547
1500	75	0.0015	537
2000	61	-0.0002	526
2500	54	-0.0002	573

S3.4 Utilizing Model and Experimental Control

We claim that varying the total input power scales the temperature profile linearly and preserves relative profiles as a function of axial position. We verify this claim by varying the input power and comparing curves for identical flow and susceptor configuration conditions, shown in Fig. S10. It is clear that for the regime in which our model is trained, the temperature profile can be linearly scaled up and down by varying the absolute input power, preserving the relative profile features. We employ this feature to predict the temperature profile of a given configuration and vary the input power to maintain the maximum temperature. When sweeping possible susceptor configurations, it is important to limit the maximum temperature, as reaction conversion is highly dependent on maximum temperature.

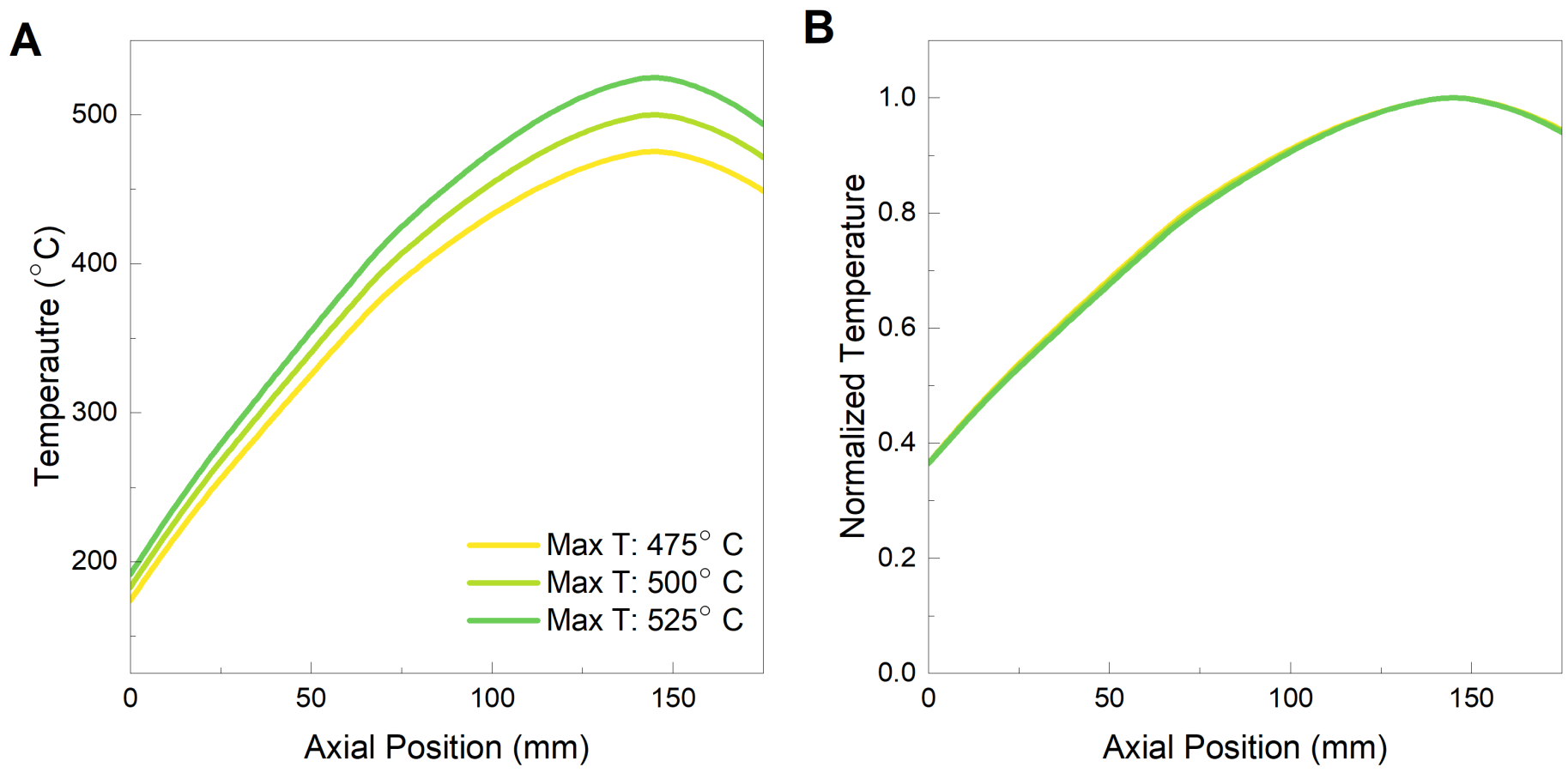

Figure S10: **Linear scaling of temperature profile with power and maximum temperature.** (**A**) We plot the axial temperature profile for a non-uniform digital susceptor at a GHSV of 2500 h^{-1} with maximum temperatures varying from 475-525°C. (**B**) We plot the same three temperature curves each normalized by maximum temperature, showing that for plots of reasonably close maximum temperature ($\sim$ 50°C) the temperature profile scales linearly up/down with maximum temperature.

In conducting the experiments, we employ a control loop to limit the power supplied by the linear amplifier by maintaining a local maximum temperature within the reactor. The multi-point fiber optic is used to measure the maximum temperature within the reactor for a given susceptor configuration and flow rate. The reaction rate is highly dependent on the local temperature within the reactor. In controlling the temperature, our goal is to find a reasonable comparison between two vastly different reactor configurations. Unlike other demonstrations, we do not make use of a pre-heater, which limits our ability to maintain a specific inlet gas temperature. One of the novelties in our design is that the heat supplied via induction heating both heats the gas and provides the energy necessary for the reaction to occur. The inlet reactor temperature is therefore dependent on the flow rate and the heating rate at the inlet. In the design of any reactor, a maximum operating temperature exists due to material limitations of the reactor walls/flanges/baffle or the catalyst itself. We therefore limit the maximum temperature within the reactor to adhere to practical design limitations and show that reactor configurations which limit the temperature gradient in the bulk of the reactor (and typically exhibit higher inlet temperatures) have higher conversion.

S3.5 Model Validation and Cross Validation

We have fit a high fidelity model and test it on unseen data. When using our model to predict temperature profiles, we set a maximum temperature and sweep the input power and inlet temperature to ensure that the inlet and outlet boundary conditions are met. This method accounts for variations in spatial power dissipation between the sections, due to inhomogeneities in lattice sections, by scaling the input power to maintain maximum temperature. We are able to get good agreement between experimental temperature profiles not used in the training process and model prediction. Fig. S11 shows the predicted and experimental temperature plots.

To further verify the validity of our parameter extraction, we fit the model using three additional sets of test/training data and calculating the R-squared and RMSE parameter for each test point. The results can be found in Table S4 which shows that additional training splits result in equally high fidelity models. Each of the training splits results in an average R-squared value around 0.95 and an RMSE value of around 15.

Table S4: Results of cross-validation

Test Point	Test 1		Test 2		Test 3		Test 4	
	R^2	RMSE	R^2	RMSE	R^2	RMSE	R^2	RMSE
1	0.99	7.8	0.98	16.2	0.98	16.0	0.97	14.3
2	0.99	8.60	0.97	14.48	0.98	12.12	0.98	13.06
3	0.94	29.22	0.93	19.69	0.90	18.79	0.99	7.84
4	0.97	14.30	0.86	29.70	0.94	18.90	0.89	27.93
5	0.98	15.48	0.98	16.51	1.00	6.45	0.71	26.01
6	0.97	14.45	0.98	13.75	0.96	14.78	0.98	16.61
7	0.96	10.77	0.95	19.97	0.96	19.50	0.96	10.86
8	0.95	16.98	0.94	15.47	0.93	22.56	0.95	15.78
9	0.98	16.72	0.94	19.61	0.97	15.13	0.98	15.40
10	0.99	12.07	0.96	14.51	0.99	7.97	0.97	13.98
Average:	0.97	14.64	0.95	17.99	0.96	15.22	0.94	16.18

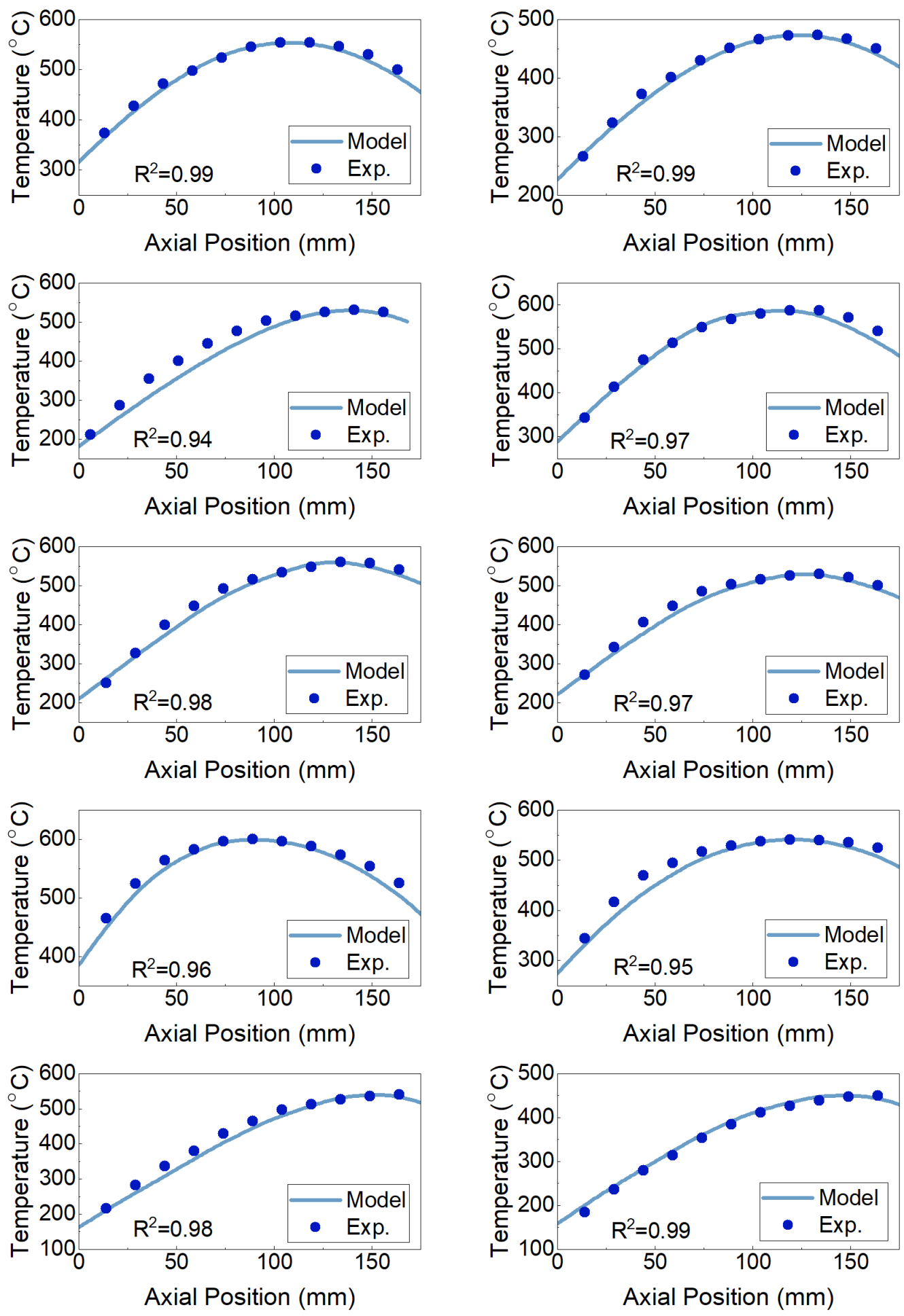

Figure S11: **Test temperature plots with model prediction**. For 10 different combinations of susceptor configuration, maximum temperature, and flow rate, the temperature profile was predicted using our 1D model trained on experimental data.

S3.6 Figure of Merit

The Figure of Merit (FOM) is used to determine how the 2^{14} possible reactor configurations should be ranked in terms of performance. This function should have a clear maximum, or minimum, and should ensure that the "best" reactor configurations are those which exhibit high conversion and minimal temperature gradients.

In Table S5 we compare the results of two different objective functions, denoted FOM_1, FOM_2. Each of these metrics were chosen such that the maximum value represents the optimal reactor configuration. FOM_1 was implemented to determine the optimal structures whose experimental results are shown in Fig. 4 of the manuscript, and is as follows,

$$\mathrm{FOM}_1 = \underset{i}{\mathrm{argmax}} \frac{\%\mathrm{CO}_{2,i} - \%\mathrm{CO}_{2,u}}{\max(\vec{T}_i) - T_{i,\mathrm{outlet}}} \tag{40}$$

where i denotes the ith reactor configuration, u denotes a uniform reactor, $\%CO_2$ is the CO_2 conversion, and $\vec{T}$ is the axial temperature profile, where $T_{i,\mathrm{outlet}}$ is the outlet temperature. FOM_2 ranks reactor configurations by predicted conversion, as follows,

$$\mathrm{FOM}_2 = \underset{i}{\mathrm{argmax}}(\%\mathrm{CO}_{2,i}) \tag{41}$$

To compare the performance of these metrics, we show the relative change in the metric for the top three reactor configurations (as determined by FOM_1) as a percent change for each of the experimental temperature and flow conditions. A value of 0 represents the optimal reactor configuration. Negative values represent percent decrease in the metric compared to the optimal value for that metric. The objective function should be chosen to select reactor configurations which display consistent increases in conversion for a wide range of temperature and flow rates, as well as clear superiority over other configurations.

FOM_1 and FOM_2 result in nearly the same optimal structures for each of the experimental conditions, as seen in Table S5, meaning that our chosen metric does select for high conversion. However, compared to FOM_2, FOM_1 results in a larger spread in the best performing structures, with percent changes on the order of 1-7 % compared to a difference of less than 1% exhibited by FOM_2. This is most likely due to the moderate increase in conversion expected at these temperatures. However, knowing that error does exist in the model, as seen in our model validation, we would like a greater distinction between the top configurations.

FOM_1 was chosen because it maximizes conversion, results in a larger spread in the top reactor configurations, and penalizes temeprature drops at the outlet. This metric was chosen to ensure that the optimal structure was clearly superior over other similar structures, as well as achieving the objectives of a high conversion, more isothermal reactor.

Table S5: Rank of top 3 reactor configurations for different metrics.

Max. Temp (°C)	GHSV (h^{-1})	Rank	Δ% FOM_1	Δ% FOM_2
500	1500	1	0	-0.95
		2	-2.01	-0.77
		3	-7.07	-2.09
	2000	1	0	-1.39
		2	-2.70	0
		3	-2.95	-2.58
	2500	1	0	0
		2	-3.97	-0.45
		3	-4.76	-2.82
525	1500	1	0	0
		2	-6.62	-0.86
		3	-7.57	-0.64
	2000	1	0	-1.12
		2	-2.4	0
		3	-3.62	-1.56
	2500	1	0	-1.57
		2	-1.14	-1.72
		3	-1.77	-1.38

S3.7 Paired T-Test

To determine whether the experimentally measured increase in conversion is statistically significant we have conducted a two-sample t-test. The T-test variable is defined as (*59*) (*60*):

$$t = \frac{\mu_d}{\sigma_d^2} \tag{42}$$

where $\mu_d = \mu_{inverse} - \mu_{uniform}$ and $\sigma_d^2 = \sigma_{inverse}^2/n + \sigma_{uniform}^2/n$, where *inverse* denotes the conversion data for an inversely designed reactor and *uniform* denotes the conversion data for a uniform reactor (as described in our experimental set up). A t value of larger than 3.55 provides a 99.9% confidence level to reject the null hypothesis and conclude the change in conversion is statistically significant.

Table S6: Results of two sample T-test

		Uniform		Inverse-Design		
Max. Temp (° C)	GHSV (h^{-1})	μ-CO_2%	σ	μ-CO_2%	σ	T-Test
500	1500	30.1	0.0218	34.2	0.2	4.5×10^4
	2000	24.4	0.0341	30.32	0.02	1.3×10^5
	2500	19.6	0.0246	26.8	0.1	2.7×10^4
525	1500	42.15	0.015	45.6	0.05	4.6×10^4
	2000	38.3	0.05	40.42	0.04	2.3×10^4
	2500	34	0.03	37.4	0.14	7.1×10^3

After performing the two-sample t-test, see Table S6, it is clear that the conversion results are statistically significant and that the increase in conversion is a result of controlling the spatial heating within the reactor.

S4 Increasing R_{AC} Ratio

S4.1 Higher AC Resistance Ratio: Modeling

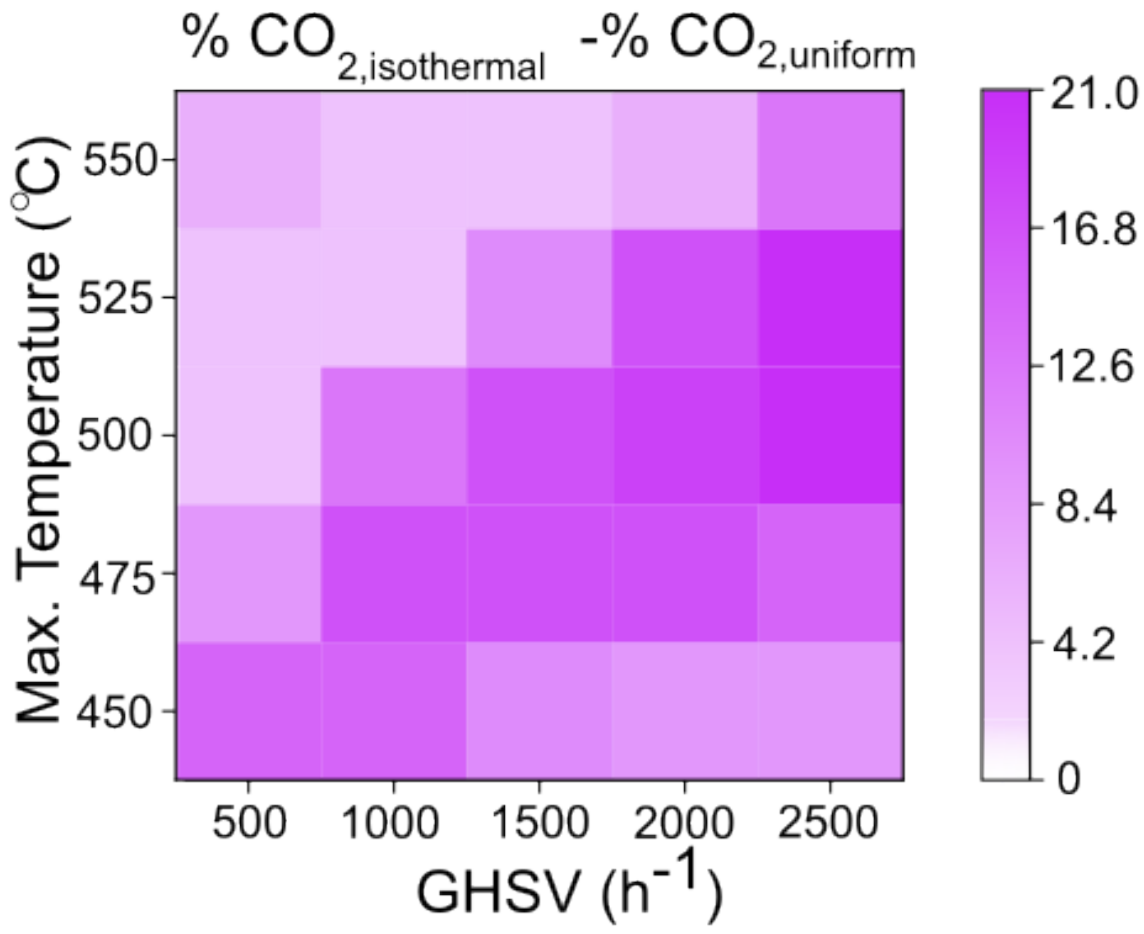

Figure S12: **Increase in conversion for reactors with ideal isothermal conditions compared to uniform susceptors.**

To demonstrate the ability to further increase conversion through axial temperature control, we explore using suscpetors with higher AC resistance ratios under the same framework as mentioned above (i.e. identical thermal conductivity, no radial heat flux) assuming first a system with perfect insulation. The theoretical maximum achievable CO_2 conversion increase occurs in an isothermal reactor. In Fig. S12 we plot the increase in CO_2 conversion between a uniformly heated susceptor and an isothermal reactor with identical geometry, with the largest increase occurring at high maximum temperatures and flow rates, consistent with the results shown in the main text, where the AC resistance ratio was 2.34. As we increase the AC resistance ratio further, so does the potential improvement in conversion.

Fig. S13a shows the increase in conversion with increasing AC resistance ratio. This increase is attributed to higher heating rates at the inlet, which increase the inlet reactor temperature, as well as maintaining a higher temperature within the reactor. In Fig. S13b we plot the simulated axial temperature profiles for AC resistance ratios of 1-∞ at a maximum temperature of 500 °C and a GHSV of 2500 h^{-1}, showing an increasingly isothermal environment with higher ratios. Much like the experimentally verified susceptors, we note that these are characterized by high heating at the inlet of the reactor, and heating at the outlet of the reactor.

Next, we consider the experimental implementation of the highest possible AC resistance ratio, which consists of sections of lattices which are non electrically conductive and therefore dissipate no power combined with sections that are electrically conductive. This creates an infinitely large contrast of their AC resistance. However, limited to the lattices available to us, which are all slightly conductive, we have to engineer a different infinite contrast susceptor. To achieve this practically, we build our reactor with sections of our high conductivity lattices filled with catalyst and sections of only catalyst (as seen in Fig. 5 of the main text). While, we recognize that the sections without

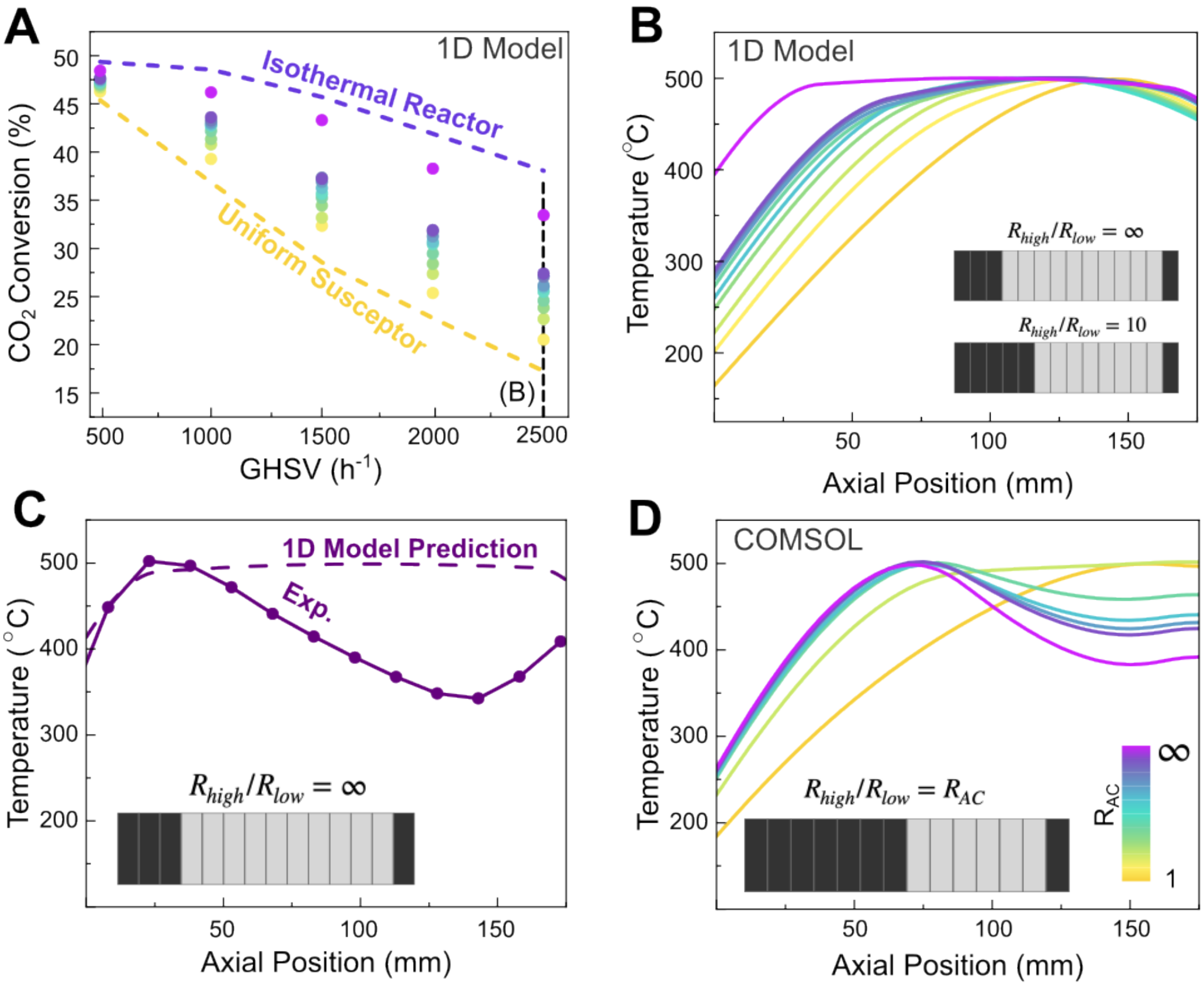

Figure S13: **Investigation of high AC resistance ratio susceptors**. (**A**) CO_2 conversion as a function of GSHV for optimized digital metamaterial reactors with varying AC resistance ratios. The maximum reactor temperature is set to 500°C. As the AC resistance ratio increases, reactor conversion approaches that of an ideal isothermal reactor. (**B**) Axial temperature profiles for reactors designed for different resistance ratios and a GSHV of 2500 h^{-1} (dashed line, (A)). The profiles become increasingly uniform with increasing AC resistance ratio. The insets show the susceptor section sequences for two different AC resistance ratios. (**C**) Comparison of the experimental (solid line with dots) and predicted (dashed line) axial temperature profiles for an inversely designed reactor assuming an infinite AC resistance ratio, as well as the ideal reactor configuration. (**D**) COMSOL simulations of the axial temperature profile for a single reactor configuration (shown) for AC resistance ratios of 1-∞. The maximum temperature was held at 500° C and the GHSV was 2500 h^{-1}. The effects of radial loss for larger R_{AC} values is apparent.

lattice will have slightly lower thermal conductivity, which we predict to be about a factor of 1.5 lower, based off the relative thermal conductivities of pure SiSiC and alumina (*37*), we believe that this structure is still a good representation of the ability to achieve a high contrast environment.

In Fig. S13c we plot the experimental axial temperature profile, the model prediction, as well as the "optimal" structure for an infinite ratio for a maximum temperature of 500° C and 2500 h^{-1}. It is clear that there is significant deviation between the predicted and experimental temperature profiles, characterized by a drop in the center of the reactor, where no heating is present. We hypothesize that the observed effects comes from non-negligible radial temperature gradients, which is further confirmed by additional multiphysics modeling in COMSOL, Fig. S13d. It is clear that radial effects are significant when the AC resistance ratio increases beyond about 2-3, making our ratio of 2.3 within this limit. It is also important to note that the extent of its effects depend on the AC resistance ratio.

It is possible to capture these radial effects by modifying our 1D pseudo-homogeneous model to account for radial heat loss. Radial heat loss can be described as a product of some heat transfer coefficient $U_{rad}(T)$ and the temperature difference from the center of the reactor to ambient. The radial loss term is then $-U_{rad}(T) \times (T - T_{\infty})$. The heat transfer coefficient is temperature dependent, of the form $U_{rad}(T) = U_{const}/T$, to capture the temperature dependent thermal conductivity of the reactor as well as the quartz tube and insulation through which heat is conducted. Using the experimental result shown above and assuming all other constants are identical, we fit this radial loss term using a least squares fit and determine that $U_{rad} = 6.8e5$. We also account for variations in thermal conductivity, as described above. Next, using the same optimization method as described in the main text, we find an optimal infinite ratio susceptor which accounts for radial temperature gradients.

S4.2 Infinite Ratio: Experimental Set Up

The experimental temperature data shown in Fig. 5c compares the temperature profile and CO_2 conversion of an inversely designed susceptor which accounts for radial losses at high AC resistance ratios and that of a "uniform" susceptor. As described in the main text and above, the infinite AC resistance ratio susceptor was constructed out of sections of high conductivity lattice and sections of only catalyst. The lack of lattice sections in parts of the reactor leads to a net higher mass of catalyst. The previously used baseline, which was 14 sections of the low-conductivity lattice, would then have a different catalyst loading than this infinite ratio structure. Therefore, we implement a different baseline for this comparison: a reactor consisting of alternating sections of lattice packed with catalyst and just catalyst, each 12.5 mm long, resulting in a reactor of near equal length and catalyst loading. As shown in section S1.6, the alternating structure has a temperature profile identical to that of a uniform susceptor.

REFERENCES

1. J. Xu, G. F. Froment, Methane steam reforming, methanation and water-gas shift: I. Intrinsic kinetics. *AIChE J.* **35**, 88–96 (1989).

2. A. M. Adris, B. B. Pruden, C. J. Lim, J. R. Grace, On the reported attempts to radically improve the performance of the steam methane reforming reactor. *Can. J. Chem. Eng.* **74**, 177–186 (1996).

3. T. Ioannides, X. E. Verykios, Development of a novel heat-integrated wall reactor for the partial oxidation of methane to synthesis gas. *Catal. Today* **46**, 71–81 (1998).

4. A. Ramirez, J. L. Hueso, M. Abian, M. U. Alzueta, R. Mallada, J. Santamaria, Escaping undesired gas-phase chemistry: Microwave-driven selectivity enhancement in heterogeneous catalytic reactors. *Sci. Adv.* **5**, eaau9000 (2019.

5. B. Maansson, B. Andresen, Optimal temperature profile for an ammonia reactor. *Ind. Eng. Chem. Proc. Des. Dev.* **25**, 59–65 (1986).

6. H. Yang, I. Nuran Zaini, R. Pan, Y. Jin, Y. Wang, L. Li, J. J. B. Caballero, Z. Shi, Y. Subasi, A. Nurdiawati, S. Wang, Y. Shen, T. Wang, Y. Wang, L. Sandström, P. G. Jönsson, W. Yang, T. Han, Distributed electrified heating for efficient hydrogen production. *Nat. Commun.* **15**, 3868 (2024).

7. M. Mokashi, A. B. Shirsath, S. Demir, A. Çelik, P. Lott, S. Tischer, O. Deutschmann, Kinetics of thermal dry reforming of methane for syngas production and solid carbon capture. *React. Chem. Eng.* **9**, 2902–2914 (2024).

8. M. Mozdzierz, G. Brus, A. Sciazko, Y. Komatsu, S. Kimijima, J. S. Szmyd, An attempt to minimize the temperature gradient along a plug-flow methane/steam reforming reactor by adopting locally controlled heating zones. *J. Phys. Conf. Ser.* **530**, 012040 (2014).

9. I. Dybkjaer, Tubular reforming and autothermal reforming of natural gas—An overview of available processes. *Fuel Process. Technol.* **42**, 85–107 (1995).

10. G. Kolb, V. Hessel, Micro-structured reactors for gas phase reactions. *Chem. Eng. J* **98**, 1–38 (2004).

11. J. R. Burns, C. Ramshaw, Development of a microreactor for chemical production. *Chem. Eng. Res. Des.* **77**, 206–211 (1999).

12. D. Pashchenko, R. Mustafin, A. Mustafina, Steam methane reforming in a microchannel reformer: Experiment, CFD-modelling and numerical study. *Energy* **237**, 121624 (2021).

13. C. G. Visconti, G. Groppi, E. Tronconi, Highly conductive "packed foams": A new concept for the intensification of strongly endo- and exo-thermic catalytic processes in compact tubular reactors. *Catal. Today* **273**, 178–186 (2016).

14. R. Xie, J. Sun, Y. Shi, J. Wei, Baffled-type thermochemical reactor for high-efficient hydrogen production by methanol steam reforming. *Int. J. Hydrogen Energy* **48**, 23425–23439 (2023).

15. A. Malhotra, W. Chen, H. Goyal, P. J. Plaza-Gonzalez, I. Julian, J. M. Catala-Civera, D. G. Vlachos, Temperature homogeneity under selective and localized microwave heating in structured flow reactors. *Ind. Eng. Chem. Res.* **60**, 6835–6847 (2021).

16. D. S. Mallapragada, Y. Dvorkin, M. A. Modestino, D. V. Esposito, W. A. Smith, B.-M. Hodge, M. P. Harold, V. M. Donnelly, A. Nuz, C. Bloomquist, K. Baker, L. C. Grabow, Y. Yan, N. N. Rajput, R. L. Hartman, E. J. Biddinger, E. S. Aydil, A. D. Taylor, Decarbonization of the chemical industry through electrification: Barriers and opportunities. *Joule* **7**, 23–41 (2023).

17. S. T. Wismann, J. S. Engbæk, S. B. Vendelbo, F. B. Bendixen, W. L. Eriksen, K. Aasberg-Petersen, C. Frandsen, I. Chorkendorff, P. M. Mortensen, Electrified methane reforming: A compact approach to greener industrial hydrogen production. *Science* **364**, 756–759 (2019).

18. C. H. Lin, C. Wan, Z. Ru, C. Cremers, P. Mohapatra, D. L. Mantle, K. Tamakuwala, A. B. Höfelmann, M. W. Kanan, J. Rivas-Davila, J. A. Fan, Electrified thermochemical reaction systems with high-frequency metamaterial reactors. *Joule* **8**, 2938–2949 (2024).

19. W. Wang, G. Tuci, C. Duong-Viet, Y. Liu, A. Rossin, L. Luconi, J.-M. Nhut, L. Nguyen-Dinh, C. Pham-Huu, G. Giambastiani, Induction heating: An enabling technology for the heat management in catalytic processes. *ACS Catal.* **9**, 7921–7935 (2019).

20. D. L. Mantle, A. B. Höfelmann, Z. Ru, P. Mohapatra, C. Wan, C. Cremers, C. H. Lin, J.-P. Shen, A. Tong, J. Rivas-Davila, J. A. Fan, Enhanced temperature cycling of particulate reactors with volumetric induction heating. *ACS Sustainable Chem. Eng.* **14**, 5922–5932 (2026).

21. C. Wan, C. Cremers, A. B. Höfelmann, Z. Ru, C. H. Lin, K. N. Tamakuwala, D. L. Man-tle, P. Mohapatra, J. Rivas-Davila, M. W. Kanan, J. A. Fan, Scale-up analysis of inductively heated metamaterial reactors. *ACS Sustainable Chem. Eng.* **14**, 5323–5331 (2026).

22. L. Luthardt, T. Raczka, K. Hurle, S. Müssig, K. Mandel, Customizable induction heating profiles: From tailored colloidally stable nanoparticles toward multi-stage heatable supraparticles. *Adv. Funct. Mater.* **35**, 2412296 (2025).

23. M. R. Almind, S. B. Vendelbo, M. F. Hansen, M. G. Vinum, C. Frandsen, P. M. Mortensen, J. S. Engbæk, Improving performance of induction-heated steam methane reforming. *Catal. Today* **342**, 13–20 (2020).

24. H. Kang, S. Lee, D. Cho, O. L. Li, D. H. Lee, Development of an induction heating reactor for rapid catalytic CF4 decomposition. *J. Ind. Eng. Chem.* **139**, 458–463 (2024).

25. Y. Yan, N. Li, Y. Pan, L. Shi, G. Xie, Z. Liu, Q. Liu, Hydrogen-rich syngas production from tobacco stem pyrolysis in an electromagnetic induction heating fluidized bed reactor. *Int. J. Hydrogen Energy* **68**, 1271–1280 (2024).

26. H. Asmat, P. Paul, F. McLaren, L. Djumas, J. Bott, M. R. Hill, A. Tanksale, Electrified reformer for syngas production–Additive manufacturing of coated microchannel monolithic reactor. *Appl. Catal. B Environ. Energy* **361**, 124640 (2025).

27. P. Wang, Y. Gao, H. Yan, Z. Yang, C. Xu, D. Tang, B. Jiang, Rapid-response electromagnetic metamaterial enabling round-the-clock electrified CO_2 methanation. *Adv. Funct. Mater.* **36**, e24133 (2026).

28. F. G. Cuevas, J. M. Montes, J. Cintas, P. Urban, Electrical conductivity and porosity relationship in metal foams. *J. Porous Mater.* **16**, 675–681 (2009).

29. K. Omata, T. Ozaki, T. Umegaki, Y. Watanabe, N. Nukui, M. Yamada, Optimization of the temperature profile of a temperature gradient reactor for DME synthesis using a simple genetic algorithm assisted by a neural network. *Energy Fuel* **17**, 836–841 (2003).

30. K. Omata, M. Yamada, Artificial neural network and grid search aided optimization of temperature profile of temperature gradient reactor for dimethyl ether synthesis from syngas. *Ind. Eng. Chem. Res.* **48**, 844–849 (2009).

31. C. S. Li, A. D. Frankhouser, M. W. Kanan, Carbonate-catalyzed reverse water-gas shift to produce gas fermentation feedstocks for renewable liquid fuel synthesis. *Phys. Sci.* **3**, 101021–3864 (2022).

32. K. N. Tamakuwala, R. P. Kennedy, C. S. Li, B. Mutz, P. Boller, S. R. Bare, M. W. Kanan, Intermediate-temperature reverse water-gas shift under process-relevant conditions catalyzed by dispersed alkali carbonates. *JACS Au* **5**, 1083–1089 (2025).

33. P. Schmidt, V. Batteiger, A. Roth, W. Weindorf, T. Raksha, Power-to-liquids as renewable fuel option for aviation: A review. *Chem. Ing. Tech.* **90**, 127–140 (2018).

34. O.-S. Joo, K.-D. Jung, I. Moon, A. Y. Rozovskii, G. I. Lin, S.-H. Han, S.-J. Uhm, Carbon dioxide hydrogenation to form methanol via a reverse-water-gas- shift reaction (the CAMERE process). *Ind. Eng. Chem. Res.* **38**, 1808–1812 (1999).

35. A. Ortona, C. D'Angelo, S. Gianella, D. Gaia, Cellular ceramics produced by rapid prototyping and replication. *Mater. Lett.*, **80**, 95–98 (2012).

36. P. Sangsuwan, J. A. Orejas, J. E. Gatica, S. N. Tewari, M. Singh, Reaction-bonded silicon carbide by reactive infiltration. *Ind. Eng. Chem. Res.* **40**, 5191–5198 (2001).

37. S. Gianella, D. Gaia, A. Ortona, High temperature applications of Si—SiC cellular ceramics. *Adv. Eng. Mater.* **14**, 1074–1081 (2012).

38. R. Lemlich, A theory for the limiting conductivity of polyhedral foam at low density. *J. Colloid Interface Sci.* **64**, 107–110 (1978).

39. A. Srinivasan, C. Depcik, One-dimensional pseudo-homogeneous packed-bed reactor modeling: I. Chemical species equation and effective diffusivity. *Chem. Eng. Technol.* **36**, 22–32 (2013).

40. J. Zhu, X. Cui, S. S. Araya, Comparison between 1D and 2D numerical models of a multi-tubular packed-bed reactor for methanol steam reforming. *Int. J. Hydrogen Energy* **47**, 22704–22719 (2022).

41. O. Levinspeil, *Chemical Reaction Engineering* (John Wiley & Sons, 3rd Ed., 1999).

42. Q. Dong, Y. Yao, S. Cheng, K. Alexopoulos, J. Gao, S. Srinivas, Y. Wang, Y. Pei, C. Zheng, A. H. Brozena, H. Zhao, X. Wang, H. E. Toraman, B. Yang, I. G. Kevrekidis, Y. Ju, D. G. Vlachos, D. Liu, L. Hu, Programmable heating and quenching for efficient thermochemical synthesis. *Nature* **605**, 470–476 (2022).

43. C. Sammoutos, A. Kitsopoulou, P. Lykas, D. Gonidaki, E. Vidalis, D. Korres, H. R. Rahbari, C. Tzivanidis, E. Bellos, Dynamic investigation of thermochemical heat upgrade and alternative industrial heating technologies. *Energies* **18**, 1990 (2025).

44. V. Rudnev, D. Loveless, R. L. Cook, *Handbook of Induction Heating* (CRC Press, Boca Raton, 2nd Ed., 2017), 772 pp.

45. C. P. Bidinosti, E. M. Chapple, M. E. Hayden, The sphere in a uniform RF field—Revisited. *Concepts Magn. Reson. Part B* **31B**, 191–202 (2007).

46. J. R. Nagel, Induced Eddy currents in simple conductive geometries: Mathematical formalism describes the excitation of electrical Eddy currents in a time-varying magnetic field. *IEEE Antennas Propag. Mag.* **60**, 81–88 (2018).

47. X. Nan, C. Sullivan, presented at the *IEEE 34th Annual Conference on Power Electronics Specialist, 2003*. PESC ‘03. (2026), vol. 2, pp. 853–860.

48. N. Dukhan, Ö. Bağcı, M. Özdemir, Experimental flow in various porous media and reconciliation of Forchheimer and Ergun relations. *Exp. Therm. Fluid Sci.* **57**, 425–433 (2014).

49. W. Zhang, K. E. Thompson, A. H. Reed, L. Beenken, Relationship between packing structure and porosity in fixed beds of equilateral cylindrical particles. *Chem. Eng. Sci.* **61**, 8060–8074 (2006).

50. E. B. E. Hassan, J. Hoffmann, Review on pressure drop through a randomly packed bed of crushed rocks. *Discov. Appl. Sci.* **6**, 3004–9261 (2024).

51. M. Ö. Çarpinlioğlu, E. Özahi, M. Y. Gündoğdu, Determination of laminar and turbulent flow ranges through vertical packed beds in terms of particle friction factors. *Adv. Powder Technol.* **20**, 515–520 (2009).

52. S. K. Sharma, S. K. Srivastava, B. S. Sharma, Thermal conductivity of solids at high temperatures and high pressures. *Int. J. Mod. Phys. B* **21**, 4419–4427 (2007).

53. H. S. Fogler, *Elements of Chemical Reaction Engineering* (Prentice Hall PTR, Upper Saddle River, NJ, 4th Ed., 2006), 1080 pp.

54. B. Nait-Ali, K. Haberko, H. Vesteghem, J. Absi, D. Smith, Thermal conductivity of highly porous zirconia. *J. Eur. Ceram. Soc.* **26**, 3567–3574 (2006).

55. F. Molaei, H. Siavoshi, Molecular dynamics studies of thermal conductivity and mechanical properties of single crystalline α-quartz. *Solid State Commun.* **320**, 114020 (2020).

56. T. L. Bergman, *Fundamentals of Heat and Mass Transfer* (John Wiley & Sons, 2011), 2249 pp.

57. D. Baillis, R. Coquard, in *Cellular and Porous Materials*, A. Öchsner, G. E. Murch, M. J. S. De Lemos, eds., (Wiley, 1st Ed., 2008), pp. 343–384.

58. D. Doermann, J. F. Sacadura, Heat transfer in open cell foam insulation. *J. Heat Transfer* **118**, 88–93 (1996).

59. M. Xu, D. Fralick, J. Z. Zheng, The differences and similarities between two-sample t-test and paired *T*-test. *Shanghai Arch. Psychiatry* **29**, 184–188 (2017).

60. C. Dougherty, *Introduction to Econometrics* (Oxford Univ. Press, Oxford, 2nd Ed., 2002).
